# Fabrication of Conjugated Conducting Polymers by Chemical Vapor Deposition (CVD) Method

**DOI:** 10.3390/nano15060452

**Published:** 2025-03-16

**Authors:** Meysam Heydari Gharahcheshmeh

**Affiliations:** Department of Mechanical Engineering, San Diego State University, 5500 Campanile Drive, San Diego, CA 92182, USA; mheydari@sdsu.edu

**Keywords:** chemical vapor deposition (CVD), oxidative CVD (oCVD), vacuum-based manufacturing, conducting polymers

## Abstract

Chemical vapor deposition (CVD) is a highly adaptable manufacturing technique used to fabricate high-quality thin films, making it essential across numerous industries. As materials fabrication processes progress, CVD has advanced to enable the precise deposition of both inorganic 2D materials, such as graphene and transition metal dichalcogenides, and high-quality polymeric thin films, offering excellent conformality and precise nanostructure control on a wide range of substrates. Conjugated conducting polymers have emerged as promising materials for next-generation electronic, optoelectronic, and energy storage devices due to their unique combination of electrical conductivity, optical transparency, ionic transport, and mechanical flexibility. Oxidative CVD (oCVD) involves the spontaneous reaction of oxidant and monomer vapors upon their adsorption onto the substrate surface, resulting in step-growth polymerization that commonly produces conducting or semiconducting polymer thin films. oCVD has gained significant attention for its ability to fabricate conjugated conducting polymers under vacuum conditions, allowing precise control over film thickness, doping levels, and nanostructure engineering. The low to moderate deposition temperature in the oCVD method enables the direct integration of conducting and semiconducting polymer thin films onto thermally sensitive substrates, including plants, paper, textiles, membranes, carbon fibers, and graphene. This review explores the fundamentals of the CVD process and vacuum-based manufacturing, while also highlighting recent advancements in the oCVD method for the fabrication of conjugated conducting and semiconducting polymer thin films.

## 1. Introduction

Conducting polymers exhibit desirable properties, such as electronic conductivity, ionic conductivity, optical transparency, and mechanical flexibility, making their fabrication into thin films essential for advancing next-generation technologies [1,2]. Potential applications of conducting polymers extend across multiple scientific and device domains, including optical displays, solar cells, electrochromic energy storage devices, catalytic processes, targeted drug delivery, tissue regeneration, memory and logic components, biomedical instruments, sensors, actuators, antistatic coatings, corrosion protection films, soft robotics, and widely accessible devices for the internet of things (IoT) [1,2,3,4,5,6]. 

Although conducting polymers offer significant potential for various applications, their integration into commercial and industrial markets remain limited. For successful commercialization, it is crucial to benchmark the performance of conducting polymer-based devices against those made with conventional materials. Their unique advantages, such as mechanical flexibility, lightweight nature, and suitability for wearable and stretchable technologies, position conducting polymers as promising candidates for integration into modern electronic and energy devices [3].

The chemical vapor deposition (CVD) method facilitates the deposition of insoluble polymers without the need for dissolution, ensuring uniform coating while preventing solvent-induced damage to solution-sensitive substrates [1,3,7,8]. Various CVD techniques have been developed to enable vapor-phase reactants to undergo polymerization mechanisms similar to those observed in solution-based synthesis. These methods include CVD polymerization through (i) chain growth initiated by free radicals or cations, primarily achieved in initiated CVD (iCVD) [9,10,11], and (ii) step-growth polymerization, which serves as the fundamental principle of oxidative CVD (oCVD) [3,7,12,13,14,15,16,17,18,19,20,21,22,23]. Step-growth polymerization, in contrast to chain-growth polymerization, involves the gradual formation of polymer chains through the reaction of multifunctional monomers. Unlike chain-growth mechanisms, where monomer addition occurs at an active site on a growing polymer chain, step-growth polymerization proceeds through the successive combination of smaller oligomers, eventually forming high-molecular-weight polymers. In oCVD, this mechanism is facilitated by the continuous exposure of monomers to a vapor-phase oxidant, allowing for controlled polymerization and thin film formation [3,12,24,25,26,27,28,29].

For most conjugated macromolecules, achieving optimal properties, such as high electrical conductivity, requires a CVD approach that replicates the step-growth polymerization mechanism. This necessity led to the development of oCVD, where vacuum chamber design, reactant vapor introduction strategies, and process conditions are specifically optimized for step-growth polymerization. The ability of oCVD to reliably fabricate conjugated conducting and semiconducting polymers with highly desirable characteristics is particularly significant for the fabrication of cutting-edge optoelectronic and renewable energy storage devices [2,12,17,22,30,31,32].

The oCVD process is a one-step approach that directly converts vapor-phase monomers and oxidant vapors into thin conjugated conducting and semiconducting polymer films [2,7,12,17,22,33,34,35,36,37,38]. At the substrate surface, step-growth polymerization and simultaneous doping occur in a single deposition step. The properties of oCVD-derived thin films can be precisely controlled by adjusting the flow rates of vapor-phase reactants [39,40], along with key process parameters, such as deposition temperature [30,34,41] and chamber pressure [12]. The oxidant saturation ratio (OSR) in the oCVD method is a critical process parameter that significantly impacts the texture and nanostructure of the deposited thin films, ultimately playing a crucial role in determining their electrical conductivity [1,39]. When multiple monomers are introduced, modifying their flow rate ratios allows for fine-tuning the composition and characteristics of the resulting copolymer films [3,11].

As a highly versatile deposition technique, oCVD offers several advantages for fabrication of conjugated conducting and semiconducting polymers, including conformal coating formation, low-temperature processing, solvent-free synthesis, uniform film growth, mechanical flexibility, scalability for industrial applications, and substrate independence [3]. Even when alternative thin film deposition methods are available, CVD is often preferred for achieving high-quality, reproducible layers essential for optoelectronic and biomedical device applications. The small-molecule reactants used in CVD can be purified to a high degree, ensuring superior material quality. Additionally, deposition in a controlled vacuum environment enhances interfacial integrity, while the all-dry processing approach eliminates the risk of residual solvents. This aspect is particularly crucial, as poor biocompatibility in polymer coatings is often linked to trapped small molecules rather than the macromolecular structure of the film itself [7,11].

The first-generation oCVD reactors use solid oxidants, such as sublimated FeCl_3_ vapor [39]. The second-generation oCVD reactors utilize liquid oxidants, such as vanadium oxytrichloride (VOCl_3_) [31,39,40], and antimony pentachloride (SbCl_5_) [34,40,42]. Liquid oxidants remove the need for post-deposition rinsing, enabling a fully dry, one-step process that is cost-effective and suitable for solvent-sensitive and temperature-sensitive substrates [12]. Water-assisted oCVD (W-A oCVD) further enhances film crystallinity by improving oxidant decomposition with water vapor [40]. The W-A oCVD process enhances monomer interaction with dopants, leading to larger crystallite formation, greater ionic dopant incorporation, and reduced backbone chlorination. Additionally, water vapor scavenges protons during oxidation, promoting higher molecular weight polymer chains through equilibrium shifts favoring dimerization [12,40]. Using the W-A oCVD method with SbCl_5_ as an oxidant, PEDOT thin films have achieved a record electrical conductivity of 7520 ± 240 S/cm by optimizing oxidant saturation and π-π stacking distances [40]. Unlike vacuum reactors using solid oxidants, which require a large crucible-to-substrate distance, liquid oxidants allow for a reduced chamber volume, resulting in higher precursor yield and improved efficiency within a more compact reactor [30].

By eliminating the need for solvents, oCVD prevents de-wetting effects that often result in pinhole defects [43]. In solution-based processing, interfacial surface energy drives de-wetting, an issue that becomes more pronounced as film thickness decreases, making it particularly difficult to achieve uniform ultrathin coating [12,13]. In contrast, oCVD enables the deposition of pinhole-free conducting polymer films as thin as 10 nm [7,12,39,44]. Furthermore, the absence of solvents ensures that the substrate remains unaffected, eliminating risks of dissolution, swelling, or structural degradation [3,12]. The oCVD process allows polymer growth on a wide range of substrates, including common and cost-effective materials, without requiring specialized surface pretreatment [12]. This contrasts with substrate-specific surface modification techniques, such as electropolymerization, which necessitates an electrically conductive substrate or self-assembled monolayers (SAMs), which only form on specific surfaces, like gold [2]. A key advantage of oCVD is its ability to operate at low temperatures, making it suitable for direct deposition on fragile substrates. such as fabrics, paper, and membranes. In contrast, deposition techniques that require high temperatures often exceed the thermal stability of the intended substrate, necessitating an additional transfer step from a heat-resistant surface to the final substrate [4,12,34,45,46]. By enabling direct low-temperature deposition, oCVD eliminates the need for complex and expensive transfer processes, simplifying fabrication and improving efficiency.

The oCVD process enables the uniform deposition of conjugated conducting and semiconducting polymer thin films on complex substrates by eliminating surface tension effects and allowing non-directional diffusion of reactants [3,12,30,42]. In contrast, liquid-based fabrication methods, such as spin-coating, face challenges in achieving conformal coverage due to issues such as liquid bridging, limited uniformity, non-conformality at low thicknesses, and delamination [13,42,43]. The elimination of surface tension effects, a major drawback in solution-based fabrication techniques, like spin-coating, is a key advantage of the oCVD method, enabling the deposition of highly conformal coatings [30]. The ability to achieve uniform, conformal coatings of conducting polymers provides significant benefits for renewable energy applications. The fabrication of conjugated conducting polymer thin films by the oCVD method preserve electrode geometry, maintain active redox sites, and enhance ion transport, which is crucial for high-performance electrochemical energy storage devices, such as supercapacitors and batteries [2,42,43]. Beyond electrochemical energy storage devices, the oCVD method’s ability to fabricate highly conformal PEDOT coatings (the most applicable conjugated conducting polymers in optoelectronic devices) as a stable and efficient hole transport layer provides a key advantage for solar cell applications [34,39]. By applying conformal coatings to artificially textured surfaces, the available surface area is increased, enhancing light absorption in the photoactive layer [12,34]. Conformal coatings are also essential for everyday materials, such as paper, textiles, foams, electrospun mats, and membranes, allowing device fabrication while preserving breathability on fiber-based substrates [3,12]. Additionally, conformality ensures complete protection and functionalization of non-planar surfaces, making it valuable for medical devices, industrial components, and printed circuit boards [3].

This review article highlights the advantages of the CVD method, with a particular focus on the oCVD process for the fabrication of conjugated conducting and semiconducting polymers. The fundamentals of the CVD process were explored in detail, including key aspects, such as the gas delivery system, vacuum reaction chamber, vacuum system, and mean free path. Additionally, critical concepts related to conductance in vacuum systems, effective pump speed, and flow regimes were discussed, providing a comprehensive understanding of vacuum-based manufacturing technologies. Furthermore, this review highlights recent advancements in the oCVD process, particularly the use of liquid oxidants, and examines the impact of deposition temperature and oxidant saturation ratio on the fabrication of high-quality conjugated conducting polymers. These insights contribute to the optimization of thin-film manufacturing techniques and expand the potential applications of vacuum-based polymer deposition techniques in next-generation electronic, optoelectronic, and energy storage devices.

## 2. Chemical Vapor Deposition (CVD) Method 

With advancements in materials fabrication, CVD technology has evolved to enable the precise deposition of both inorganic 2D materials, such as graphene [4,47], transition metal dichalcogenides (TMDs) [4,48,49,50,51,52], and high-quality polymeric thin films [3,7,12,53]. This progress offers exceptional conformality and precise nanostructure control across a diverse range of substrates. The large-scale fabrication of uniform conducting polymers using high-throughput roll-to-roll (R2R) processing is crucial for industrial applications. To facilitate this, chemical vapor deposition (CVD) and other vapor deposition techniques present promising solutions for accelerating the commercialization of conjugated conducting polymers in mass production [3,4,12]. CVD is a widely utilized technique for material processing, where thin films are fabricated on a heated substrate through chemical reactions of gaseous precursors [12]. Unlike physical vapor deposition (PVD) techniques (including thermal evaporation, electron beam (E-Beam) evaporation, direct current (DC) sputtering, radio frequency (RF) sputtering, magnetron sputtering), the CVD method relies on chemical reactions to enable tunable deposition rates and fabricate high-quality thin films with superior conformality. The growing demand for conjugated conducting polymers, organic semiconductors, and inorganic semiconductor thin films, played a crucial role in accelerating the development of CVD technology [3,54]. As the semiconductor industry advanced, the necessity for high-purity, defect-free, and precisely engineered thin films became paramount to enhance device performance and reliability [4,55].

CVD emerged as a transformative deposition method, offering superior conformality, precise thickness control, and the ability to tailor material properties at the atomic level [3,4,11,12]. Its versatility has been instrumental in shaping modern microelectronics, enabling the fabrication of integrated circuits, transistors, and nanostructured materials essential for next-generation computing, optoelectronics, flexible electronics, photonic devices, advanced energy systems, and quantum technologies. Strong vacuum pumps, like ion pumps (also known as ion getter pumps), can create an ultra-high vacuum (UHV) in the range of 10^−6^–10^−9^ Torr, or even an extreme high vacuum (XHV) below 10^−9^ Torr. In contrast, the CVD method does not require such extreme vacuum conditions, and it typically operates at process pressures in the range of 100 m Torr to 1 Torr, making it a widely used technique in electronics, optoelectronics, surface modification, and biomedical applications. 

The adaptability of the CVD method enables precise control over material properties and structural characteristics, making it indispensable for next-generation electronic and optoelectronic applications. To meet the increasing demands of modern technologies, various specialized CVD techniques have been developed, each tailored for specific applications. Plasma-enhanced CVD (PECVD) enhances reaction kinetics and allows low-temperature processing, making it well-suited for temperature-sensitive substrates by providing plasma, which is made of ionized, high-energy gases [4,56]. In PECVD, plasma is generated using direct current (DC), radio-frequency (RF) voltage, or microwave sources, which are coupled to the reactor, leading to a significant reduction in the reaction temperature [4]. Metal organic CVD (MOCVD) involves the use of metal–organic precursors, typically volatile liquids, which are vaporized to form thin films. The MOCVD method is essential for depositing III–V compound semiconductors (comprised of elements from groups III and V of the periodic table) [4,57,58], high-temperature superconductors [59,60,61,62,63], and epitaxial layers [4], making it particularly valuable for the fabrication of advanced electronic and optoelectronic devices.

Oxidative CVD (oCVD) relies on the spontaneous reaction between oxidant and monomer vapors upon adsorption to the substrate, producing step-growth polymerization that typically yields conducting or semiconducting polymer films [3,12,30,43]. oCVD facilitates the fabrication of conjugated conducting and semiconducting polymers, offering new possibilities for flexible electronics and organic optoelectronics [3,12,13]. The initiated CVD (iCVD) method employs vapor-phase reactants, including an initiator and monomers, which undergo chain-growth polymerization upon adsorption onto a temperature-controlled substrate [9,64]. Utilizing an initiator allows for significantly lower filament temperatures, preserving the monomer’s organic functional groups. This retention of functional groups enables precise control over the film’s wettability and surface reactivity [11]. Meanwhile, iCVD enables the deposition of functional polymer films with controlled thickness and composition, expanding the use of CVD in surface coatings, biomedical applications, and nanostructured materials [4,9,11]. These advancements significantly enhance CVD’s capabilities, allowing for atomic-scale precision, high-quality film growth, and scalable manufacturing for next-generation devices. Other variations of CVD include photo-assisted CVD and laser-assisted CVD, where high-intensity light or a laser is used to stimulate and enhance the deposition process, promoting thin film formation [4].

The most common CVD configuration is the horizontal tube reactor, where substrates are positioned horizontally, vertically, or at a tilt to optimize gas flow. Vertical reactors often use showerhead mixers for improved material uniformity and growth rates [4]. In hot-wall CVD reactors, the entire vacuum chamber is uniformly heated using a heating filament or external furnace, whereas in cold-wall CVD reactors, only the substrate and its surrounding area are heated while the reactor walls remain cold, enabling rapid heating and cooling. Hot filament CVD (HFCVD) uses resistively heated filaments, often made from tungsten, tantalum, or molybdenum, to thermally decompose precursors that deposit onto cooler substrates, typically forming inorganic films, like amorphous silicon or silicon nitride [4,65,66,67].

The deposition rates in CVD techniques are highly influenced by process parameters, such as chamber pressure, precursor flow rates, and deposition temperature, as well as the specific approach used, such as low-pressure CVD (LPCVD), and PECVD. Typically, deposition rates in CVD methods range from 1 to 10 nm/min [30]. Lower deposition rates are associated with the formation of high-quality, highly conformal thin films, whereas higher deposition rates facilitate faster production but may compromise film quality. Table 1 provides a summary of the common precursors utilized and functional materials fabricated in various CVD methods. It outlines the typical precursor combinations used in different CVD techniques.

### 2.1. Fundamental of CVD Method

**Figure 1:**
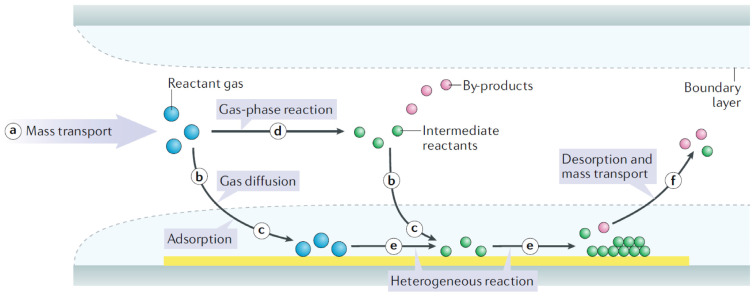
Schematic illustration of the CVD process. (**a**) Reactant gases enter the reactor. (**b**,**c**) Gases either diffuse through the boundary layer and adsorb onto the substrate or (**d**) undergo gas-phase reactions, forming intermediates and by-products, which then deposit via diffusion and adsorption. (**e**) Surface diffusion and heterogeneous reactions occur, leading to thin film formation. (**f**) Unreacted species and by-products desorb and exit as exhaust [4]. Reproduced with permission [4]. Copyright 2021, Nature.

Despite the diversity of CVD techniques, the fundamental process remains consistent and follows several key steps, as illustrated in Figure 1 [4]. Initially, reactant gases are delivered into the vacuum reactor chamber, where their flow rate is regulated through a mass flow controller (MFC) or a needle valve to ensure precise mass transport (Figure 1a). The reactant gases may either diffuse across the boundary layer to reach the substrate surface, enabling gas diffusion (Figure 1b) or undergo gas-phase reactions, leading to the formation of intermediate reactants and gaseous by-products (Figure 1d). In both scenarios, the reactant gases and intermediate reactants adsorb onto the heated substrate’s surface and spread across it (Figure 1c). The subsequent reactions at the gas–solid interface result in the steady formation of a thin film through processes of heterogenous nucleation and growth (Figure 1e). Eventually, any gaseous by-products and unreacted species desorb from the substrate’s surface and are evacuated from the reaction area (Figure 1f). Gas-phase reactions typically occur when temperatures are sufficiently high or when additional energy, like plasma, is applied. It is important to highlight that heterogeneous nucleation and growth play a critical role when deposition relies on surface catalysis by the substrate [4].

Heterogeneous nucleation and growth are a key process in the formation of thin films, particularly in the CVD method, where a reaction occurs at the interface between two distinct phases, typically between the gas phase and a solid substrate [1,4]. During heterogeneous nucleation, gas-phase reactants undergo surface processes, such as adsorption and surface diffusion, before nucleating as solid-phase clusters on the substrate. These nucleation sites serve as initial points for further growth, eventually forming a continuous thin film. The characteristics of the heterogenous nucleation and growth process depend on factors like substrate temperature, precursor properties, and surface energy, which collectively influence thin film morphology, grain size, and uniformity.

The growth of thin films during heterogeneous nucleation can occur through various mechanisms, including island growth (also known as Volmer–Weber grow), layer-by-layer growth (also known as Frank–van der Merwe growth), and layer-plus-island growth modes [85,86]. Island growth occurs when atoms or molecules aggregate into isolated clusters on the substrate surface due to stronger interactions between deposited atoms compared to their interactions with the substrate, resulting in discrete island formation [86]. In contrast, layer-by-layer growth involves the sequential addition of atomic or molecular layers on the substrate surface, driven by stronger interactions between the film atoms and the substrate compared to interactions between adjacent film atoms. A combination of these mechanisms, known as layer-plus-island growth, involves initial layer formation followed by island growth, creating a more complex film morphology [86,87].

Achieving desired thin film characteristics involves challenges, such as minimizing defects, ensuring uniformity, and optimizing grain size, as defects, like dislocations, voids, and grain boundaries, can degrade electrical, mechanical, and optical properties [87]. Uniform growth is critical for consistent performance, while grain size affects conductivity, crystallinity, and overall behavior. Therefore, careful optimization of parameters, like substrate temperature, precursor flux, and deposition time, is essential to produce high-quality thin films for applications in microelectronics, optoelectronics, and energy devices.

The CVD system must fulfill several essential requirements to ensure efficient and controlled high quality thin film deposition. First, it must enable the precise and adjustable delivery of gas-phase reactants, allowing for accurate control over film composition and growth rates. Second a well-sealed vacuum reaction chamber with a leak rate below 10 mTorr/min is crucial for maintaining a stable environment, minimizing contamination, and ensuring precise control over reaction pressure, which directly influences thin film morphology, uniformity, and overall material quality [12]. Furthermore, the system must incorporate a reliable energy source, such as thermal, plasma, or optical excitation, to drive the chemical reactions essential for thin film deposition [4,12,88]. Proper exhaust gas treatment through the use of an appropriate vacuum pump is essential to safely neutralize oxidants and gaseous by-products while effectively removing them from the chemical reaction zone to maintain process stability and prevent contamination. Lastly, an advanced automatic process control system is required to enhance deposition stability, reproducibility, and efficiency, reducing human intervention while optimizing thin film characteristics. By meeting these fundamental criteria, a CVD system can achieve precise and scalable material synthesis for various industrial and research applications.

### 2.2. Gas Delivery System in the CVD Method

The volatile gaseous reactants used in the CVD processes are typically stored in high-pressure gas cylinders, which are placed in temperature-controlled environments at a constant negative pressure [4]. The gas supply system begins at the outlet of these high-pressure cylinders, where mechanical pressure regulators are installed to precisely control the output pressure before the gases are transported through the delivery lines to the reaction vacuum chamber. To regulate the mass flow rate of reactant gases into the reactor, various types of valves are integrated into the delivery line, including ball valves for quick shut-off, needle valves for fine flow adjustments, and pneumatic valves for automated control in high-precision applications. The vacuum coupling radiation (VCR) gaskets are commonly used in the gas delivery system to ensure leak-free, high-purity connections between components, maintaining system integrity and preventing contamination during thin film deposition.

In CVD processes, gas throughput (Q) plays a critical role in controlling reaction conditions, ensuring uniform thin film deposition, and maintaining process stability. The gas throughput is typically measured in standard cubic centimeters per minute (SCCM), where 1 SCCM corresponds to 1 cm^3^ of gas at a standard temperature and pressure (0 °C and 1 atm (760 Torr)). The relationship between gas throughput (Q), system pressure (P), and vacuum pump speed (S) is expressed by the following equation [89,90]:

(1) Q=P·S

where Q represents the gas throughput in a unit of Torr·L/s, P is the system pressure in a unit of Torr, and S  is the vacuum pump speed, measured in L/s.

Proper control over throughput ensures stable deposition conditions, and maintaining a steady gas influx and exhaust helps prevent unwanted pressure fluctuations that may lead to thin film inhomogeneity. In practical applications, Torr·L/s is often converted to SCCM for easier gas flow measurements and adjustments. The approximate conversion factor is: Torr·L/s≈78.9 SCMM. By carefully regulating gas throughput, engineers and researchers can fine-tune deposition parameters to achieve high-quality thin films with desired composition, thickness uniformity, and structural integrity.

Mass flow controllers (MFCs) play a critical role in regulating gas delivery to the reactor chamber by automatically adjusting the gas flow rate in the SCCM through feedback control based on the mass of the gas in transit. For liquid-phase precursors, a carrier gas is typically employed to transport the vaporized precursor through a bubbling process [12,30,40]. In contrast, solid precursors with low volatility are introduced into the reaction chamber either by dissolving them in a suitable solvent for vaporization or by sublimating them directly from a crucible into the gas phase, ensuring precise and efficient delivery for thin film deposition [4,12].

### 2.3. Vacuum Reaction Chamber in CVD Method

The two primary configurations of the vacuum reaction chamber in CVD systems are horizontal and vertical designs, each suited for different deposition requirements and scalability [4]. Horizontal reaction chambers are commonly used for batch processing, where substrates are placed on a boat inside a quartz tube and exposed to the reactant gases. Vertical reaction chambers are preferred in high-throughput processes, such as semiconductor wafer fabrication, where substrates are loaded onto susceptor plates or rotating platforms to ensure uniform gas exposure. 

In CVD reactors, stainless steel 304 (SS 304) and 316 (SS 316) are commonly used for the chamber walls due to their excellent corrosion resistance, thermal stability, and mechanical strength. The reaction chamber is also constructed from quartz, a material widely employed in tube shape reactors in semiconductor manufacturing due to its high-temperature tolerance and resistance to rapid thermal cycling. The chamber is connected to a gas inlet injector via a metal flange, which is often equipped with cooling components to prevent thermal damage and maintain system integrity. To ensure laminar gas flow and uniform precursor distribution, a gas distributor with precision-engineered through-holes is typically integrated into the system [11,90].

The CVD systems may incorporate hot-wall or cold-wall reactor designs. In hot-wall reactors, the chamber walls are heated to maintain a uniform temperature distribution, reducing unwanted deposition on chamber surfaces. In contrast, cold-wall reactors use localized heating for the substrate to minimize contamination and improve deposition selectivity on the substrate. The substrate is typically placed on a substrate holder, commonly referred to as a boat or susceptor, which is designed to withstand the high temperatures and reactive environments of the CVD process. These holders are generally made from quartz or graphite, materials known for their excellent chemical stability, thermal resistance, and minimal interaction with the deposited films [4,91,92]. In high-temperature applications, particularly in MOCVD epitaxial growth processes, Inconel (a nickel-based superalloy), is also used due to its superior oxidation resistance, mechanical strength, and durability under extreme thermal conditions [59,60,62,63,78].

### 2.4. Heating Source in the CVD Method

In the CVD process, the heating source is a crucial component that drives the chemical reactions necessary for thin film formation on the substrate. The heating source must supply adequate thermal energy to decompose precursor gases into reactive species, promoting their reaction and subsequent deposition as a solid thin film [4,11,12]. The choice and configuration of the heating source significantly affect the deposition rate, thin film morphology, crystallinity, and overall material characteristics. Common heating sources in CVD include resistive heating, induction heating, infrared (IR) heating, and plasma-assisted methods, each offering distinct advantages based on specific application needs [4]. For instance, resistive heating is widely favored due to its ability to deliver uniform heat distribution across the substrate, while induction heating provides rapid temperature control, making it ideal for processes requiring elevated temperatures. Various thermocouples (such as types B, J, K, R, and S) are employed for precise temperature monitoring in different operational environments.

In plasma-enhanced CVD (PECVD), plasma generated through electrical discharge in a gaseous medium (e.g., argon) serves as an energy source, enabling thin film deposition at lower temperatures, which is particularly advantageous for temperature-sensitive substrates, like flexible electronics [4]. Notably, oCVD and iCVD techniques have enabled the fabrication of conducting and functional polymers at significantly lower deposition temperatures, ranging from room temperature to 150 °C, making them ideal for applications involving temperature-sensitive and flexible substrates while broadening their applicability across various fields [3,12,30,42]. The deposition temperature in various CVD methods is highly dependent on the specific technique and the materials being fabricated. For instance, metal organic chemical vapor deposition (MOCVD), commonly used in the fabrication of high-temperature superconductors, typically requires deposition temperatures in the range of 700 °C to 1000 °C [59,60,61,62,63,77,78]. Additionally, the geometry and materials of the substrate holder influence heat transfer efficiency. Radiant heating techniques involving halide lamps, electric induction, or lasers can be utilized to selectively heat the substrate. In cylindrical CVD reactors, commonly known as furnace reactors, resistance heating systems featuring three distinct heating zones and aluminum silicate insulating cotton are often employed to ensure a uniform temperature field over an extended length. This uniformity is crucial for consistent film quality and reproducibility in high-performance applications [4].

### 2.5. Vacuum System and Mean Free Path

Purging the deposition vacuum chamber to start the deposition process and obtaining the necessary pressure for transport of the reactants relies on the vacuum system, where measurement and control of the vacuum are essential and complement one another. The primary reasons for utilizing a vacuum system in the fabrication of functional materials through CVD methods are to ensure a contamination-free process and to achieve a high mean free path for precursor vapors [4]. In a vacuum environment, the mean free path, which is defined as the average distance that a vapor molecule travels before colliding with another molecule, is significantly increased due to the reduced gas density. This minimizes gas-phase scattering, thereby reducing unwanted side reactions and improving the uniformity and purity of the deposited thin film. Additionally, a higher mean free path allows precursor molecules to reach the substrate surface more efficiently, leading to an enhanced deposition rate and improved film quality, which is crucial for applications in electronics, optoelectronics, and advanced material coatings. 

The behavior of low-pressure gases is generally described by kinetic gas theory, which treats gases as a system of individual molecules with negligible intermolecular van der Waals forces, allowing their motion and interactions to be analyzed statistically. According to kinetic gas theory, gas molecules travel at thermal velocity, determined by temperature, and undergo collisions that define their motion. There is an inverse relation between the process pressure in the vacuum reactor and mean free path (λ), as in the following equation [93,94]:

(2) $$\lambda =\frac{k_{B}T}{\sqrt{2}\pi d^{2}p}$$

where $k_{B}$ represents Boltzmann’s constant, T denotes the absolute temperature, d is the diameter of the gas molecule, and p refers to the reactor pressure during deposition.

To accurately measure the pressure within a vacuum system, a variety of pressure transducer gauges are employed, each offering unique measurement principles and advantages. Among the most commonly used mechanical gauges are piezo sensors, capacitance manometers, and diaphragm manometers, which detect vacuum levels by measuring physical changes in strain or electrical capacitance. While the Bourdon gauge is a cost-effective and durable option, it lacks an electronic output, making it unsuitable for automated feedback control systems. In contrast, the capacitance manometer is a widely preferred choice due to its high accuracy and broad measurement range, typically spanning four orders of magnitude. The capacitance manometer gauge is highly versatile, as it provides precise readings for nearly any gas and allows seamless integration with electronic displays and automated feedback controllers, enabling real-time pressure monitoring and process optimization in CVD and other vacuum-based deposition systems. 

The operating pressure during thin film deposition is precisely maintained and regulated using a throttle valve, which continuously adjusts in response to real-time pressure readings from a capacitance manometer gauge [3,12]. This dynamic feedback mechanism ensures stable process pressure throughout the deposition process, optimizing film uniformity and quality.

Vacuum pumps serve as the primary mechanism for generating vacuum conditions and facilitating mass transport within a system. Different types of vacuum pumps operate across a range of vacuum levels, classified as follows:

(i) Rough vacuum: Pressure range of 760 Torr to 1 mTorr;
(ii) Medium vacuum: Pressure range of 1 mTorr to 10^−6^ Torr;
(iii) High vacuum: Pressure range of 10^−6^ to 10^−9^ Torr;
(iv) Ultra-high vacuum (UHV): Pressure range of 10^−9^ to 10^−12^ Torr;
(v) Extreme high vacuum (XHV): Pressure range of below than 10^−12^ Torr;
(vi) Rough vacuum conditions can be achieved using a variety of pumps, including rotary piston mechanical pumps, dry mechanical pumps, sorption pumps, and blower/booster pumps, each suited for different industrial and laboratory applications. For high vacuum environments, turbo molecular pumps, cryogenic pumps, and diffusion pumps are commonly utilized, providing efficient gas removal and maintaining stable vacuum levels. Achieving an ultra-high vacuum requires specialized pumps, such as ion pumps, which rely on ionization and electrostatic trapping to remove residual gas molecules, making them essential for high-precision applications, like space research, and particle accelerators. In a CVD system, mechanical pumps are sufficient for many processes and can achieve vacuum levels down to ≈1 mTorr. While oil-sealed pumps are more cost-effective compared to dry pumps of similar capacity, they introduce potential contamination due to oil back streaming, which may affect film purity and process stability. For systems with large reaction vacuum chambers, a rotary pump (with a pumping speed ranging from 0.5 to 325 L/s) combined with a Roots pump (capable of 50 to 35,000 L/s) is an effective solution for rapid gas evacuation, enhancing process efficiency and maintaining stable vacuum conditions [4].

### 2.6. Pump-Down in Vacuum Systems

The pump-down process in a vacuum system refers to the gradual reduction of pressure as gases are removed from the reactor chamber. Initially, the pressure drop follows an exponential decay, as governed by the equation [95]:

(3) $$p\left(t\right)=p_{0}e^{\frac{-S}{V}t}$$

where p (t) is the pressure at time t, $p_{0}\;$denotes the initial pressure (which is atmospheric pressure ≈760 Torr), S signifies the pump speed in unit of L/s, and v is the volume of reactor chamber in liters.

During the initial pump down process, gas molecules are rapidly evacuated from the reactor chamber, leading to a sharp pressure drop. However, as the pressure decreases, the effect of outgassing from chamber walls and internal components becomes more significant, slowing down the pump-down rate. Outgassing, which includes desorption of water vapor and volatile contaminants, becomes the dominant factor in achieving high or ultra-high vacuum conditions. To minimize the effect of outgassing, techniques such as baking the reactor chamber, plasma cleaning, or using cryogenic traps are often employed in the vacuum systems.

### 2.7. Conductance in Vacuum Systems

In vacuum technology, conductance refers to the ability of a vacuum component or system to facilitate the flow of gas. Conductance is a key parameter that influences the efficiency of vacuum pumping and gas transport within the system [96]. Conductance can be considered the inverse of flow resistance, meaning that higher conductance allows gases to move more freely, while lower conductance restricts flow. Conductance (C) is defined as the ratio of gas throughput or molecular flux (Q) to the pressure drop ($∆P=P_{1}-P_{2}$) across a vacuum system [90]:

(4) $$C=\frac{Q}{\left(P_{1}-P_{2}\right)}$$

where *C* is conductance (typically in Liters per Second, L/s), *Q* is the molecular flux or gas throughput (Torr·L/s), and $P_{1}-P_{2}\;$ is the pressure drop between reactor side and vacuum pump side in the unit of Torr.

### 2.8. Effective Pump Speed in Vacuum Systems

The effective pump speed in vacuum technology is a critical parameter that determines the rate of gas removal from the vacuum reactor chamber and the ability to maintain the desired pressure in a CVD reactor. The effective pump speed ($S_{effective}$) is defined as the actual pumping speed at the reactor chamber and is influenced by both the intrinsic/nominal pumping speed (S) of the vacuum pump and the conductance of the connecting vacuum lines. The effective pump speed ($S_{effective})$ is generally lower than the nominal pump speed (S) due to gas flow restrictions and pressure drops in the connecting filter trap, vacuum tubing, roughing valve, and throttle valve. As a result, optimizing the vacuum system layout is crucial for achieving efficient gas removal and maintaining stable deposition conditions in CVD processes.

The conductance of the vacuum tube connecting the vacuum reactor to the vacuum pump plays a key role in determining the effective pump speed and the relationship between the effective pump speed ($S_{effective})$, nominal pump speed (S), and conductance (C) is given by the following equation [97]:

(5) $$\frac{1}{S_{effective}}=\frac{1}{S}+\frac{1}{C}$$

As can be noted from the above equation, the low conductance in the vacuum line significantly reduces the effective pumping speed, limiting the gas removal rate from the vacuum reactor. To enhance pumping efficiency, shorter and wider vacuum tubes with smooth inner surfaces should be used to minimize flow resistance. 

### 2.9. Flow Regimes in the Vacuum Systems

In general, to enhance the conductance of the vacuum system, larger diameter vacuum tubing, shorter pump lines, and high-conductance valves are often used. Proper system design ensures that vacuum pumps operate efficiently, leading to improved process stability and thin film quality. It is important to note that conductance in a vacuum system varies significantly depending on the flow regime, which can be categorized into viscous flow, molecular flow, and transition flow. The classification of flow regimes in the vacuum system is determined by the Knudsen number ($K_{n}$), which describes the relationship between the mean free path (λ) of gas molecules and the smallest characteristic dimension of the vacuum system’s geometry (L), as described by the following definition [98]:

(6) $$K_{n}=\frac{\lambda }{L}=\frac{k_{B}T}{\sqrt{2}\pi d^{2}p\;L}$$

where $k_{B}$ is the Boltzmann constant, T represents the temperature, d denotes the diameter of the gas molecule, and p is the pressure of the system.

Flow regimes in the vacuum system are classified as follows [99]:

(i) Molecular flow ($K_{n}>1):\;$Gas molecules move independently, with collisions occurring more frequently with chamber walls than with other molecules, typical in high and ultra-high vacuum and high mean free path.
(ii) Viscous flow ($K_{n}<0.01$): Gas molecules interact predominantly with each other, behaving like a continuous fluid, common at higher pressures and low mean free path.
(iii) Transition flow (${0.01<K}_{n}<1$): Intermediate regime where both molecular and viscous flow characteristics are present, occurring in medium vacuum conditions.

Under atmospheric pressure, gas diffusion in mesoporous materials predominantly occurs in the Knudsen regime, where the mean free path of gas molecules is comparable to or larger than the pore size, leading to molecular collisions with the pore walls rather than intermolecular collisions. In contrast, the ultra-high vacuum diffusion setup enables the generation of significantly high Knudsen numbers at macroscopic scales by operating under ultra-high vacuum conditions, where molecular flow is dominated by wall interactions rather than bulk gas dynamics [100].

## 3. Methods for the Fabrication of Conjugated Conducting and Semiconducting Polymers 

Conjugated conducting and semiconducting polymers are classified based on charge carrier polarity as p-type (hole-dominant), n-type (electron-dominant), or ambipolar (both carriers) [5,6]. In p-type doping, an oxidizing agent extracts an electron from the highest occupied molecular orbital (HOMO) of the polymer matrix, leaving behind a positive hole along the conjugated backbone. In contrast, n-type doping involves a reducing agent injecting an electron into the lowest occupied molecular orbital (LUMO) of a polymer matrix, resulting in a negative charge carrier along the conjugated polymer chain [2,5,101,102]. Ionization energy (IE) and electron affinity (EA) are key parameters that determine p-type and n-type doping characteristics in semiconductors. For p-type doping, the EA of the acceptor dopant must be equal to or greater than the IE of the organic polymer host, facilitating electron transfer from the HOMO of the polymer to the LUMO of the dopant. In other words, p-type doping occurs when the LUMO of the dopant is positioned near or below the HOMO of the polymer host [5]. In n-type doping, the HOMO of the dopant is positioned near or above the LUMO of the organic polymer host [5,6,103].

**Figure 2:**
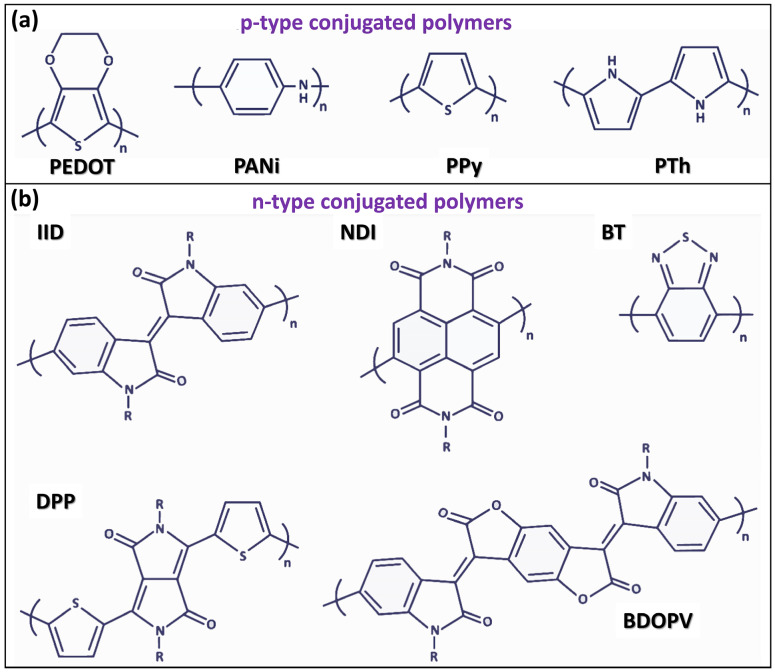
Chemical structures of key p-type and n-type conducting and semiconducting polymers. (**a**) Structures of widely studied p-type conjugated polymers, including PEDOT, PANI, PPy, and PTh [6]. (**b**) Structures of prominent n-type conjugated polymers, such as IID, NDI, BT, and DPP BDOPV [6]. (**a**,**b**) Reproduced with permission [6]. Copyright 2022, MDPI.

Among p-type conjugated conducting polymers, the most widely studied and applicable are poly(3,4-ethylenedioxythiophene) (PEDOT) [30,34,40,42,43,104,105,106], polyaniline (PANI) [6,17,107,108], polypyrrole (PPy) [2,109,110], polythiophene (PT) [18,29,111], and their derivatives. In contrast, high-mobility n-type conjugated conducting polymers are relatively rare, unstable under ambient conditions, and prone to oxidation [5,6]. The most reported n-type conjugated conducting polymers include naphthalene diimide (NDI), benzodifurandione-based oligo(p-phenylene vinylene) (BDOPV), diketopyrrolopyrrole (DPP), isoindigo (IID), and benzothiadiazole (BT) [2,5]. The chemical structures of the most widely used p-type and n-type conjugated conducting and semiconducting polymers are shown in Figure 2a and Figure 2b, respectively [6].

**Figure 3:**
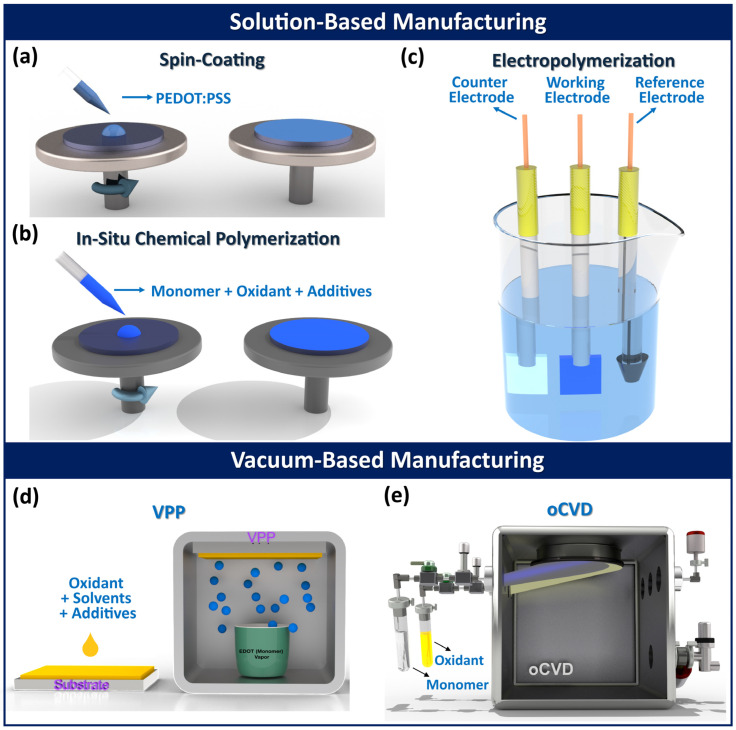
Overview of common fabrication methods for conjugated conducting polymers**.** Solution-based techniques include (**a**) spin-coating [3], (**b**) in situ chemical polymerization (ICP) [2], and (**c**) electrochemical polymerization [2], while vapor-based methods consist of (**d**) vapor phase polymerization (VPP) [2], and (**e**) oxidative chemical vapor deposition (oCVD) [39]. (**a**) Reproduced with permission [3]. Copyright 2019, Wiley. (**b**–**d**) Reproduced with permission [2]. Copyright 2024, Royal Society of Chemistry. (**e**) Reproduced with permission [39]. Copyright 2019, Science.

While the CVD method is a highly versatile manufacturing technique for fabricating high quality conducting polymers, semiconducting polymers, and functional polymers, particularly through approaches, like oCVD [3,7,12,30,42,112] and iCVD [9,10,11,64,88,113,114], other fabrication techniques are also exhibited in Figure 3 [2]. Solution-based methods utilize liquid-phase processing techniques, such as spin-coating [115,116], in situ chemical polymerization (ICP) [117,118,119], and electropolymerization [120,121]. In contrast, vapor-phase deposition methods enable polymerization from gaseous reactants, including vapor phase polymerization (VPP) [122,123,124,125], and oCVD [3,11,12,14,33,42,43,54,126,127,128,129,130]. Additionally, alternative polymerization strategies, like hydrothermal synthesis [131], electrospinning [132], self-assembly [133], interfacial polymerization [134], and plasma polymerization [135], have been explored for fabricating conducting polymer thin films and composites, broadening their application potential.

Spin-coating is a common solution-based method for depositing polymer thin films and involves four steps: (i) dispensing the solution, (ii) spinning the substrate, (iii) removing excess solution, and (iv) thermal annealing (Figure 3a) [2]. Polymeric thin film quality in spin-coating depends on factors like spin speed, polymer molecular weight, viscosity, solvent volatility, and surface interactions [2,136]. Electrical conductivity in spin-coated conducting polymer films improves with high-boiling-point polar solvents, such as dimethyl sulfoxide (DMSO), N,N-dimethyl formamide (DMF), N,N-dimethyl acetamide (DMAc), sorbitol, glycerol, ethylene glycol (EG), and dichloromethane (DMC) [2,5,6,137]. In in situ chemical polymerization (ICP), a monomer, oxidant, and additives are spin-coated onto a substrate, enabling simultaneous polymerization and doping (Figure 3b) [2]. Properties of conjugated polymer thin films fabricated by the ICP depend on molecular weight, oxidant type, solvent, additives, and morphology, while oxidant characteristics influence oxidation rate, affecting film structure and conductivity [2,138]. Electropolymerization deposits conjugated conducting polymer films on a conductive electrode via anodic oxidation in a three-electrode setup (Figure 3c) [2]. Applying a deposition potential oxidizes the monomer, forming radical cations that polymerize into oligomers, leading to conjugated polymer thin film formation [2,139]. Counterions integrate into the polymer, influencing conductivity in electropolymerization. Solvent and electrolyte selection are crucial for ionic conductivity and stability [2,140,141].

Vapor phase polymerization (VPP) is a two-step process for fabricating conducting polymer films, where a low-volatility oxidant is first applied via spin-coating, dried, and then exposed to monomer vapor in a vacuum chamber, initiating polymerization at the oxidant–monomer interface (Figure 3d) [2]. VPP operates at an ambient or moderate vacuum (5–30 Torr), while recently, vacuum-assisted methods have become common [2,125,142,143]. The polymerization rate in VPP can be adjusted using polar organic solvents (e.g., DMSO, NMP, DMF) and chelating ligands (e.g., EDTA) to modify oxidant reactivity [2,5,6,144]. Base inhibitors (e.g., imidazole, PEI, and pyridine) raise the pH, minimize side reactions, and improve the conductivity of fabricated conjugated polymers by the VPP method [145,146,147]. The use of surfactants (e.g., PEG-PPG-PEG) improve electrical properties of conducting polymers fabricated by the VPP method through controlling the polymerization rate and preventing oxidant crystallization [148]. Key fabricated factors in the VPP method include monomer exposure time, deposition pressure, deposition temperature, humidity, oxidant acidity, and post-deposition acidic rinsing treatments [2].

The oCVD method is a solvent-free and scalable manufacturing technique for depositing conducting and semiconducting polymers as highly conformal thin films, with tunable nanostructures achieved by engineering dopant levels and process parameters [4,7,12,13,15,30,33,34,40,42,43,53]. In the oCVD process, a monomer and oxidant are delivered as vapors into a hot-wall vacuum chamber, where the oxidant vapor initiates step-growth polymerization on a temperature-regulated substrate, resulting in the formation of high-quality conductive polymer films (Figure 3e) [2]. The oCVD method enables direct polymerization of vapor-phase monomers and oxidants in a controlled vacuum environment, offering a single-step, truly dry process. Key parameters in the oCVD method, such as oxidant saturation ratio (OSR) [39,40], deposition temperature [25,30,41,42] chamber pressure [3,11,12,149], choice of oxidant [2,12,39], and water-assisted growth techniques [33,40], tailor properties of fabricated conducting polymers. The oCVD method is largely substrate independent, enabling deposition of conjugated conducting polymers on a wide range of surfaces, including organic and inorganic, planar and non-planar, as well as porous and dense materials, while forming covalently grafted films without the need for linker molecules [2,3,12]. Unlike solution-based techniques, oCVD offers solvent-free processing, low-temperature deposition, and scalability, making it well-suited for roll-to-roll (R2R) production of large-area, high-performance conducting polymer thin films [4,11,12,54]. The highest reported electrical conductivity of 7520 ± 240 S/cm in PEDOT thin films was achieved using the oCVD method with water-assisted growth and SbCl_5_ as an oxidant, resulting in excellent optoelectronic properties [40].

The key advantages and disadvantages of each fabrication method for conjugated polymers have been summarized based on recent literature [2,3,7,12,53,125,136,146,149,150,151,152,153] and are provided in Table 2. Various factors, including quality of thin film, conformality, substrate compatibility, and process scalability have been considered to assess their suitability for applications in electronic, optoelectronic, and energy-related devices. In general, the oCVD method is a highly versatile manufacturing technique for fabricating ultrathin, highly conformal coatings of conducting and semiconducting polymers, enabling large-scale production through roll-to-roll (R2R) processing [3,7,12,53].

Unlike solution-based methods, oCVD is a solvent-free technique that minimizes the generation of hazardous liquid waste. However, the choice of oxidant significantly influences the process’s overall environmental footprint. Recent advancements in oCVD, particularly the use of liquid oxidants, such as SbCl_5_ and VOCl_3_, along with optimized process parameters (mainly oxidant saturation ratio and deposition temperature) enable the fabrication of conductive polymers with minimal oxidant by-products. This development transforms oCVD into a true single-step, dry process, eliminating the need for acidic rinsing post-treatment steps. Additionally, any volatile by-products can be effectively controlled through appropriate exhaust and filtration systems.

## 4. Oxidative Chemical Vapor Deposition (oCVD) Method 

**Figure 4:**
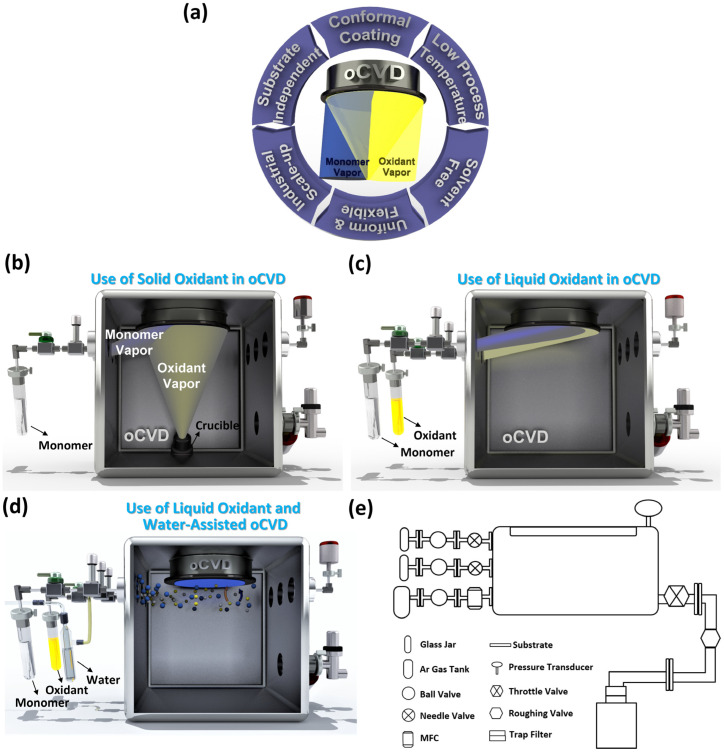
Schematic representation of oCVD reactors. (**a**) Key features and benefits of the oCVD method for fabricating conjugated conducting polymers [3]. The oCVD process using (**b**) a solid oxidant [39], (**c**) a liquid oxidant [39], and (**d**) a liquid oxidant with water-assisted growth [40]. (**e**) Diagram illustrating the oCVD setup with a reduced reactor volume enabled by liquid oxidants, including dedicated delivery lines for monomers and oxidant species, along with exhaust configurations [30]. (**a**) Reproduced with permission [3]. Copyright 2019, Wiley. (**b**,**c**) Reproduced with permission [39]. Copyright 2019, Science. (**d**) Reproduced with permission [40]. Copyright 2021, Wiley. (**e**) Reproduced with permission [30]. Copyright 2024, Wiley.

The oCVD method has emerged as a highly effective roll-to-roll (R2R) manufacturing technique, enabling the simultaneous polymerization, doping, and thin film formation to achieve conformal coatings on complex nanostructured substrates [12]. Its low to moderate deposition temperature allows the direct integration of conducting and semiconducting polymer thin films onto thermally sensitive substrates, such as plants [154], papers [7,155], textiles [112], membranes [156], carbon fibers [13,42,43], and graphene [1,15]. For commercial-scale applications, the oCVD technique offers the advantage of fabricating uniform, defect-free, and ultra-thin (<100 nm) conjugated conducting polymers over large-area substrates (>600 cm^2^), making it a promising method for scalable manufacturing [3,12,112]. As a versatile deposition method, oCVD provides key benefits for fabricating conjugated conducting and semiconducting polymers, such as conformal coatings, low-temperature processing, solvent-free synthesis, uniform film growth, mechanical flexibility, scalability, and substrate independence (Figure 4a) [3]. Even when alternative deposition techniques exist, CVD is often favored for producing high-quality, reproducible layers critical for optoelectronic and biomedical devices.

First-generation oCVD reactors use solid oxidants, like sublimated FeCl_3_ vapor (Figure 4b) [39], while second-generation reactors employ liquid oxidants, such as VOCl_3_ and SbCl_5_, enabling a fully dry, cost-effective one-step process suitable for solvent-sensitive and temperature-sensitive substrates (Figure 4c) [39]. Water-assisted oCVD (W-A oCVD) enhances thin film crystallinity by reducing the π-π stacking distance through improved oxidant decomposition and increased monomer–dopant interactions, achieving a record electrical conductivity of 7520 ± 240 S/cm for PEDOT thin films fabricated with the SbCl_5_ oxidant (Figure 4d) [40]. In contrast to vacuum reactors that use solid oxidants and require a large crucible-to-substrate distance, liquid oxidants enable a reduced chamber volume, leading to higher precursor yield and greater efficiency in a more compact reactor design, as shown in Figure 4e [30].

**Figure 5:**
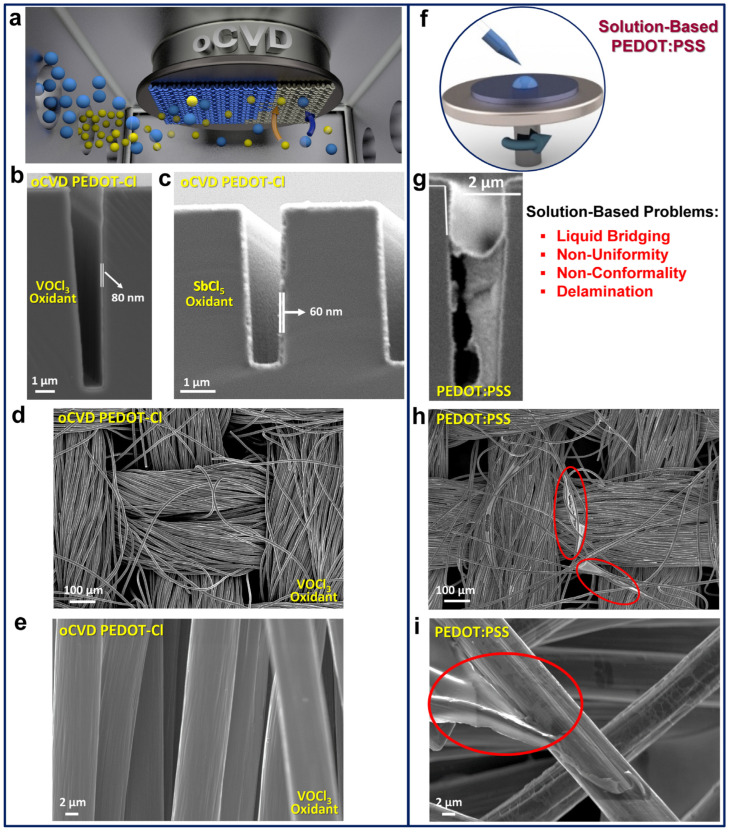
High Conformality of Conjugated Polymers Fabricated by the oCVD Method. (**a**) Schematic of the oCVD process, where monomer vapor (represented by blue spheres) and oxidant vapor (represented by yellow spheres) are introduced into a vacuum chamber [43]. Ultrathin, highly conformal PEDOT thin films deposited via oCVD on silicon trench wafers using (**b**) VOCl_3_ [39], and (**c**) SbCl_5_ oxidants [34]. (**d**,**e**) Highly conformal oCVD PEDOT coatings on carbon cloth electrodes for redox flow batteries [43]. (**f**) Schematic of the spin-coating method [3]. (**g**) Spin-coating method demonstrating its non-uniform coverage on silicon trench wafers [130]. (**h**,**i**) SEM images of PEDOT:PSS on carbon cloths show issues with spin-coating, including liquid bridging, non-uniformity, poor conformality, and delamination due to surface tension effects [43]. (**a**,**d**,**e**,**h**,**i**) Reproduced with permission [43]. Copyright 2020, Wiley. (**b**) Reproduced with permission [39]. Copyright 2019, Science. (**c**) Reproduced with permission [34]. Copyright 2024, ACS. (**f**) Reproduced with permission [3]. Copyright 2019, Wiley. (**g**) Reproduced with permission [130]. Copyright 2008, ACS.

The oCVD process allows uniform deposition of conjugated conducting and semiconducting polymer thin films on complex substrates by removing surface tension effects and enabling non-directional reactant diffusion (Figure 5a–e) [34,39,43]. Unlike liquid-based methods, like spin-coating, which face issues, like liquid bridging, poor uniformity, and delamination (Figure 5f,i) [3,43], oCVD’s key advantage lies in its ability to fabricate highly conformal coatings, overcoming these limitations [30]. Achieving uniform, conformal coatings of conducting polymers offers substantial advantages for renewable energy applications. The oCVD method enables the fabrication of thin films of conjugated conducting polymers that preserve electrode geometry, sustain active redox sites, and enhance ion transport, which is essential for high-performance electrochemical energy storage devices such as supercapacitors and batteries [2,12,42,43]. In addition, the oCVD process’s ability to produce highly conformal PEDOT coatings, a widely used conjugated polymer in optoelectronics, offers a crucial advantage as a stable and efficient hole transport layer in solar cell applications, where high surface area on trenched substrates enhances light absorption by the photoactive layer [34,39].

In the oCVD process, a monomer (e.g., EDOT) and an oxidant (e.g., SbCl_5_) are introduced as vapors into a hot-wall vacuum chamber, where the oxidant vapor triggers step-growth polymerization on a temperature-controlled stage, forming high-quality conductive polymer films [12,30,34,43]. Deposition rates in CVD methods vary widely depending on process parameters such as chamber pressure, precursor flow rates, and deposition temperature, as well as the specific technique employed, including low-pressure CVD (LPCVD), plasma-enhanced CVD (PECVD), and oxidative CVD (oCVD). The deposition rates typically range from 1 to 10 nm/min in the oCVD method [30]. Lower deposition rates (e.g., in the range of 1–2 nm/min) produce high-quality, highly conformal thin films, while higher rates enable faster production, often at the expense of film quality [30]. Unlike solution-based methods, oCVD processes eliminate surface tension effects, preventing pinhole formation in ultra-thin polymer films, even below 10 nm [3,4,39].

The key factor for the commercial viability of conjugated conducting and semiconducting polymers fabricated by the oCVD method is the ability to produce high-quality thin films at low deposition temperatures, ensuring compatibility with various plastic substrates. Simultaneously, there is a strong demand for a one-step, fully dry process to minimize fabrication costs [12]. Flexible substrates such as polyethylene terephthalate (PET), polyethylene naphthalate (PEN), and their blends (PEN/PET) can tolerate temperatures up to approximately 150 °C [30,157]. Optimizing oCVD parameters, such as oxidant and monomer flow rates, allows the fabrication of PEDOT films using SbCl_5_ without the need for post-deposition acidic treatment, making it well-suited for flexible, temperature-sensitive, and solution-sensitive substrates [12]. Additionally, incorporating water vapor during deposition further enhances the performance of oCVD-deposited conducting polymers while maintaining a fully dry fabrication process [40].

### 4.1. Monomers Used in the oCVD Method

So far, oCVD-based homo-polymerization and co-polymerization have been successfully demonstrated for over a dozen monomers, highlighting the versatility of vapor-phase fabrication for conjugated conducting and semiconducting polymers. A key requirement for oCVD monomers is sufficient volatility, ensuring their transition into the vapor phase for effective deposition [3,54]. Unlike solution-based methods, oCVD eliminates the need for solubility-enhancing functional groups, as neither the monomers nor the resulting polymers require dissolution [3]. The elimination of solubility requirements for monomers enables the direct polymerization of unsubstituted monomers, such as thiophene [35,111], selenophene [158], and aniline [107], all of which are liquid at ambient conditions, allowing their efficient integration into the oCVD process [3].

Modified monomers have been investigated to enhance the properties of conjugated conducting polymers fabricated by the oCVD method, particularly for bandgap tuning [3,39]. Specifically, substituting thiophene at the 3- and 4-positions restricts polymerization to the 2- and 5-positions, enabling the selective formation of linear polymer chains with high stereoregularity, which enhances π-conjugation, thereby improving charge transport efficiency and electrical conductivity [3,159]. The first successfully demonstrated conjugated polymer fabricated by the oCVD method was synthesized from 3,4-ethylenedioxythiophene (EDOT) [159], which continues to be the most extensively studied oCVD chemistry to this day [3,30,34,42].

The majority of monomers utilized in the oCVD method for fabricating conjugated conducting and semiconducting polymers exist as liquids at ambient conditions [3]. However, it is important to note that solid-state monomers (such as 3,4-propylenedioxythiophene, 2,2-dimethyl-3,4-propylenedioxythiophene, 2,2′-bithiophene, 2′:5′3″-terthiophene, and thieno 3,2-b thiophene(TT)) have also been successfully employed in oCVD polymerization, demonstrating the versatility of this deposition technique [3,160]. The incorporation of functional groups into conjugated polymer thin films fabricated via the oCVD method has been successfully achieved using various monomers, enabling post-deposition functionalization with nanoparticles or biomolecules, a feature particularly advantageous for chemical sensing, biochemical detection, and smart biomaterials applications [36].

### 4.2. Oxidants Used in the oCVD Method

In the oCVD method, step-growth polymerization is facilitated by an oxidant, typically an evaporated metal halogen salt or halogen gas [3,11,12]. Delivering oxidants, particularly solid-phase oxidants, into the oCVD reactor via the vapor phase presents a greater challenge compared to monomer vapor delivery, primarily due to the low volatility of many solid oxidants [3]. Commonly used solid oxidants in the oCVD method include iron(III) chloride (FeCl_3_) [2,13,29,31,37,39,40,54,129,161,162,163] and copper(II) chloride (CuCl_2_) [2,3,130]. Conducting polymers deposited via the oCVD method using CuCl_2_ as an oxidant displayed a porous structure, primarily due to the lower oxidizing power of CuCl_2_, which affects the polymerization process and film formation [3]. The effectiveness of an oxidant in the polymerization process is directly influenced by its standard electrode potential, which determines its ability to facilitate electron transfer through step-growth polymerization [3,162]. CuCl_2_, with a standard electrode potential (also known as the redox potential of oxidant) of 0.33 V, acts as a weaker oxidant in comparison to FeCl_3_, which has a higher standard electrode potential of 0.77 V, leading to differences in oxidation efficiency and film morphology [3,162].

A tall reactor is commonly utilized in the oCVD technique to facilitate the sublimation of solid oxidants, such as FeCl_3_, within the vacuum chamber [3,11,12,161]. Within the oCVD reactor, a heated crucible sublimes the solid oxidant, directing it toward the downward-facing substrate surface [12]. To ensure the complete removal of unreacted FeCl_3_ and its oxidation by-products, a post-deposition acidic rinsing step is typically performed, involving rinsing with hydrochloric acid (HCl), sulfuric acid (H_2_SO_4_), and methanol (MeOH) [2,12,30]. The acidic treatment effectively dissolves residual FeCl_3_ while also eliminating any undesired oxidant by-products that affect the electrical conductivity and structural integrity of conducting polymers. It is important to note that the sticking probability of the oxidant, which is influenced by its volatility, plays a crucial role in determining the conformality of the deposited conjugated polymer thin films fabricated by the oCVD method. For instance, conjugated polymer thin films fabricated by the oCVD method using bromine (Br) gas as an oxidant exhibit higher conformality compared to films produced using evaporated solid oxidants, such as FeCl_3_ [3]. 

More recently, liquid oxidants reported for the oCVD process include vanadium oxytrichloride (VOCl_3_) [2,12,15,39,43] and antimony pentachloride (SbCl_5_) [12,30,33,40,42]. Liquid oxidants are often preferred over solid counterparts in the oCVD method due to their higher volatility, improved flow control, and minimal residue formation, eliminating the need for post-deposition acidic rinsing [3,12,39]. The properties of conjugated conducting and semiconducting polymers fabricated via oCVD can be tailored by adjusting key process parameters, such as oxidant saturation ratio (OSR) [39,40], deposition temperature [25,30,41,42], chamber pressure [2,3,11,12], water-assisted growth techniques [12,33,40], and the choice of oxidant [3,12,39]. The effectiveness of an oxidant in the oCVD process is primarily determined by its volatility, thermal stability, and ability to drive the polymerization reaction forward [2,12,164]. The variation in oxidizing strength influences polymerization kinetics, film density, and the overall structural characteristics of the resulting conducting polymer, resulting in distinct morphological differences depending on the chosen oxidizing agent [3]. A summary of the commonly utilized oxidants in different fabrication processes for the conjugated conducting polymers is presented in Table 3. 

### 4.3. Step-Growth Polymerization Mechanism in the oCVD Method

**Figure 6:**
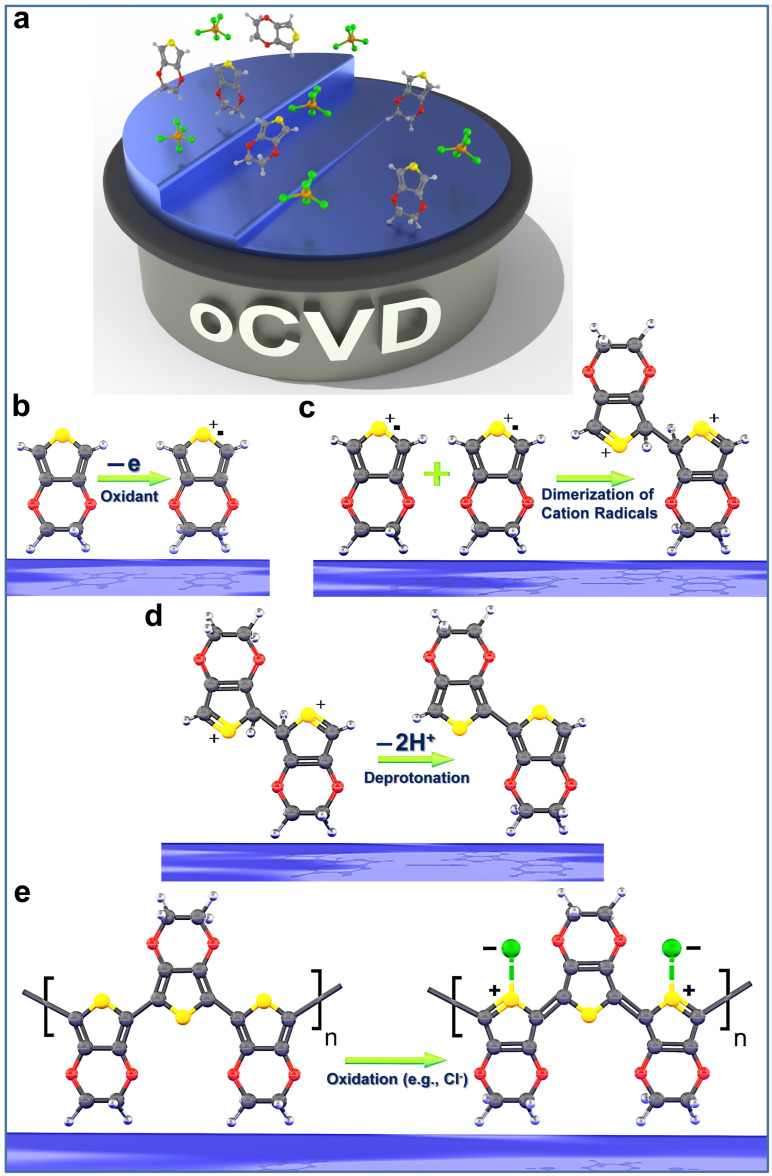
Schematic representation of step-growth polymerization in the oCVD process. (**a**) Vapor phases of the monomer (e.g., EDOT) and oxidant (e.g., SbCl_5_) are introduced onto a temperature-controlled substrate [12]. The polymerization follows a sequential process: (**b**) Oxidation of the EDOT monomer to generate radical cations, (**c**) Formation of dimers through radical coupling, (**d**) Deprotonation of the dimer to establish conjugation, and (**e**) Incorporation of counterion dopants into the PEDOT backbone. The spheres in different colors represent specific elements: dark gray for carbon, yellow for sulfur, red for oxygen, white for hydrogen, and green for chlorine [12]. (**a**–**e**) Reproduced with permission [12]. Copyright 2024, Springer.

In the oCVD process, vapor phases of monomer and oxidant species are introduced into the deposition chamber, where they are directed onto a temperature-controlled substrate, as illustrated in Figure 6a [12]. Upon contact of precursor vapors with the substrate, adsorption occurs, followed by spontaneous surface polymerization reactions. This eliminates the need for external excitation sources, such as plasma or photonic energy, allowing the polymerization to proceed efficiently under controlled conditions [3,12]. 

The kinetics of polymerization in oCVD involves multiple sequential steps, including the adsorption of monomer and oxidant vapors onto the substrate, their surface diffusion, heterogeneous nucleation, and subsequent film growth through step-growth polymerization [53]. The polymerization rate (deposition rate) is significantly influenced by key parameters, such as oxidant concentration (also known as the oxidant saturation ratio), monomer reactivity, and deposition temperature, all of which collectively determine the growth rate, molecular weight distribution, and uniformity of the polymer film [12]. Higher deposition temperatures generally result in a lower deposition rate [30,34], while an increased oxidant concentration (or equivalently, a higher oxidant saturation ratio), leads to an increased deposition rate in conductive polymers fabricated by the oCVD method [12,39]. While higher oxidant concentrations accelerate the reaction rate by enhancing monomer oxidation, an excessive amount of oxidant vapors, quantified by the oxidant saturation ratio, can induce overoxidation and conjugated backbone chlorination, disrupting the semi-crystalline characteristics and degrading charge carrier mobility and overall electrical conductivity [40,42].

From a thermodynamic perspective, the polymerization reaction in oCVD is believed to be governed by the Gibbs free energy change (ΔG), which dictates whether the reaction occurs spontaneously (∆G=∆H−T∆S, where, ΔH is the enthalpy change, T is the absolute temperature, and ΔS is the entropy change). For a favorable polymerization process, Δ*G* should be negative. The redox potential of the oxidant ($E_{ox}$), which reflects its tendency to gain electrons and thereby its ability to oxidize the monomer, plays a crucial role in determining the feasibility of monomer oxidation [3,162]. Generally, the oxidant’s redox potential must be sufficiently high to effectively accept electrons from the monomer, ensuring efficient oxidation and subsequent polymerization. Furthermore, the enthalpy change (Δ*H*) associated with bond dissociation and formation impacts the exothermic or endothermic nature of the reaction, while entropy (Δ*S*) accounts for the disorder introduced in the deposited thin film during vapor-phase polymerization. 

The formation of polymer thin films in oCVD follows a step-growth polymerization mechanism, which occurs through the following sequential steps [3,12]:

(1) Oxidation of Monomer: The oxidizing agent reacts with the EDOT monomer, generating radical cations through electron transfer (Figure 6b) [12]. This initiates the polymerization process by creating highly reactive species.
(2) Dimerization: The radical cations undergo coupling, leading to the formation of stable dimer structures (Figure 6c) [12]. This step is crucial for chain elongation and determines the growth kinetics of the polymer film.
(3) Proton Scavenging and Dimer Stabilization: The oxidizing agent’s anions act as proton scavengers, neutralizing excess protons from the dimer and stabilizing the newly formed structure (Figure 6d) [12]. This step enhances polymer integrity and prevents unwanted side reactions.
(4) Propagation: The oxidizing agent continues to react with the stabilized dimer, producing additional radical cations. This process repeats continuously, allowing the polymer chains to grow in a controlled manner across the substrate surface.
(5) Doping and Structural Stabilization: Some positively charged heterocyclic rings within the polymer backbone undergo stabilization through interactions with counter-ion dopants. This final step enhances the electrical conductivity and structural integrity of the polymer film, completing the polymerization process (Figure 6e) [12].

The doping process (exhibited in Figure 6e), results in the delocalization of charges along the conjugated polymer backbone, enabling efficient charge transport and facilitating electrical conductivity. The degree of conductivity in the polymer is directly influenced by the extent of charge delocalization and the integration of counter-ion dopants within the structure [5,12]. The counter-ion dopants stabilize the positive charges on the polymer chains, effectively modulating the electronic properties of the conjugated conducting polymers with the p-type semiconducting characteristics [30]. Unlike chain-growth polymerization, where molecular weight gradually increases as monomers are added sequentially, step-growth polymerization in the oCVD method follows an exponential progression [11]. The step-growth polymerization occurs through sequential coupling reactions, leading to the formation of progressively larger molecular units—dimers, tetramers, octomers, and eventually high-molecular-weight 2N-mers [3,11,12]. The step-growth polymerization mechanism enables the rapid formation of long polymer chains, resulting in thin films with well-defined morphology and enhanced conductivity.

A key theoretical assumption in PEDOT polymerization is that the maximum theoretical dopant-to-monomer unit ratio is 1:3, meaning one dopant ion is incorporated for every three monomer units [1,3,11,12,165]. However, experimental evidence has revealed deviations from this theoretical limit, with some reports indicating heavily doped PEDOT conjugated backbones, where the dopant-to-monomer unit ratio reaches 1:2 [12,39,40,42]. This increased doping level suggests enhanced charge carrier density, which can significantly impact the electrical properties and stability of the polymer, making it crucial to optimize doping conditions for specific applications.

### 4.4. Precursor Flow Rates and Partial Pressure in the oCVD Method

Achieving precise control over the flow rates of monomers and oxidants in the oCVD system is crucial for optimizing deposition conditions, ensuring uniform thin-film formation, and maintaining process stability. In the oCVD method, regulating the flow rates of monomer and oxidant species differs from the conventional use of mass flow controllers (MFCs) designed for standard gases. Since dedicated MFCs for reactive species used in the oCVD method are not always available, an alternative approach to flow rate control and extraction is implemented during the deposition process. Converting the reactant flow rate into standard cubic centimeters per minute (SCCM) requires degassing the oCVD reactor and applying fundamental principles of flow regulation and vacuum deposition design. The initial step in calculating the flow rates of precursor vapors in SCCM for the oCVD method is defining the reactor volume. The reactor volume (*V*) is determined using the well-known Boyle–Mariotte law ${(P_{1}V}_{1}=P_{2}V_{2})$, which establishes the relationship between pressure and volume under ideal gas conditions within the reactor [12]. Once the oCVD reactor volume is determined, the flow rates of monomer and oxidant precursor vapors can be derived in SCCM by analyzing the rate of vapor pressure change over time (dP/dt), as described in the following equation [30,39,40]:

(7) $$SCCM=60\times \left\{\left(\frac{V}{P}\right)\times \left(\frac{dP}{dt}\right)\times \frac{T_{1}}{T_{2}}\right\}-Reactor\;Leak\;Rate$$

where V represents the reactor volume, P  is the pressure recorded by the pressure transducer, dP/dt  denotes the rate of vapor pressure change over time for the reactants, $T_{1}\;$ is set at 0 °C (273.15 K) as a reference temperature, while $T_{2}$ denotes to the temperature of the inner wall of the vacuum reactor.

Once the monomer and oxidant flow rates are determined in SCCM units and the deposition pressure is known, the partial pressure of each reactant can be calculated. The partial pressure represents the individual contribution of each species to the total pressure within the deposition chamber, providing essential insight into the reactant concentration and its effect on thin film growth dynamics [12]. The partial pressure of the reactant species can be calculated using the following equation [12]:

(8) $$P_{partial\;(specific\;reactant)}=\frac{Flow\;Rate\;of\;Specific\;Reactant\;(SCCM)}{Total\;Flow\;Rate\;of\;Reactants\;(SCCM)}\times Depsotion\;Pressure$$

The above equation ensures that the proportion of oxidant and monomer in the reaction environment is accurately defined, which is critical for controlling oxidation levels, doping efficiency, and overall thin film properties. 

### 4.5. Saturation Precursor of Reactant Species in the oCVD Method

The saturation pressure ($P_{sat}$) of the monomer and oxidant species at a given deposition temperature can be calculated using the Clausius–Clapeyron equation [12,39,40]:

(9) $$P_{sat}=P_{1}\cdot \exp\left(\frac{-∆H_{vap}}{R}\left(\frac{1}{T_{2}}-\frac{1}{T_{1}}\right)\right)$$

where $P_{1}$ signifies the known pressure of reactants at approximately room temperature, $∆H_{vap}$ represents the enthalpy of vaporization, and R  denotes for the gas constant.

The Clausius–Clapeyron equation describes the relationship between temperature and vapor pressure, providing insights into how reactants transition between liquid or solid phases and vapor phases under controlled thermal conditions. By applying the Clausius–Clapeyron equation, one can determine the vapor pressure required for efficient reactant delivery, ensuring an optimal concentration of precursor species in the reaction chamber. This is particularly crucial in the oCVD process, where the precise control of monomer and oxidant vapor pressures directly influences thin film growth rates, deposition uniformity, and polymerization efficiency. Additionally, understanding saturation pressure helps in adjusting process parameters, such as chamber pressure and temperature, to maintain stable and reproducible thin-film fabrication [12].

**Figure 7:**
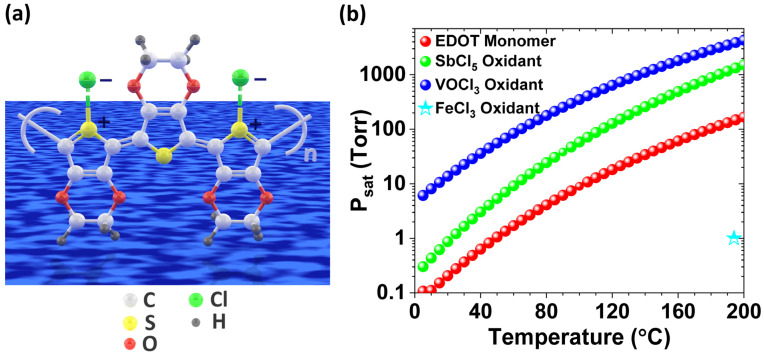
(**a**) Chemical structure of PEDOT-Cl thin films fabricated via the oCVD method, serving as a p-type conductor [30]. The structure consists of a positively charged conjugated PEDOT backbone stabilized by chloride (Cl^−^) counterions, enabling efficient hole transport. (**b**) Saturation pressure ($P_{sat}$) values of various oxidants and the EDOT monomer used in the oCVD process [12]. The plotted $P_{sat}$ values illustrate their dependence on deposition temperature, providing insights into the volatility of each reactant. (**a**) Reproduced with permission [30]. Copyright 2024, Wiley. (**b**) Reproduced with permission [12]. Copyright 2024, Springer.

PEDOT is one of the most widely used conjugated conducting polymers for energy devices due to its excellent electrical and mechanical properties [1,5,6,43,165]. PEDOT-Cl thin films fabricated via the oCVD technique differ significantly from solution-processed PEDOT:PSS films, primarily in their counterion dopants. Unlike PEDOT:PSS, which contains polystyrene sulfonate (PSS) as a counterion, oCVD PEDOT-Cl films utilize chloride ions (Cl^−^) as dopants, which effectively induce p-type conductivity, as illustrated in Figure 7a [30]. The structure of PEDOT-Cl thin films consists of a positively charged conjugated PEDOT backbone, stabilized by negatively charged chloride counterions. This p-type doping mechanism results in hole-dominated charge transport, where holes serve as the primary charge carriers responsible for conductivity [3,30]. In contrast, PEDOT:PSS solutions exhibit high acidity, with a pH ranging from 1 to 2, which can pose challenges for long-term device stability [30,34,39,166]. This acidic nature can lead to substrate degradation and compatibility issues in certain electronic applications, making oCVD PEDOT-Cl films a more stable alternative for advanced energy storage and optoelectronic devices [34,39].

For the fabrication of PEDOT thin films using the oCVD method, the vapor phase of both the monomer (EDOT) and oxidants must be introduced into the vacuum reactor chamber under controlled conditions. The enthalpies of vaporization for EDOT, SbCl_5_, and VOCl_3_ are 42.9 kJ/mol [12,39,40], 47.8 kJ/mol [40], and 6.78 kJ/mol [39], respectively, indicating the energy required for their phase transition from liquid or solid to vapor. At 25 °C, the vapor pressures of EDOT and SbCl_5_ are 0.278 Torr and 1.2 Torr, respectively [12,40]. In contrast, VOCl_3_ exhibits significantly higher volatility, with a vapor pressure of 13.8 Torr at 20 °C, making it a more readily vaporized oxidant [39]. The saturation pressure ($P_{sat}$) values for EDOT and different oxidants across various temperatures are depicted in Figure 7b [12], providing a comparative understanding of their volatility and suitability for vapor-phase deposition in the oCVD process. 

The solid oxidant FeCl_3_ has an extremely low saturation pressure at room temperature, only reaching 1 Torr near its sublimation temperature of 194 °C [40]. Managing the oxidant saturation ratio (OSR) becomes more complex when dealing with either highly volatile liquid oxidants (e.g., VOCl_3_) or low-volatility solid oxidants (e.g., FeCl_3_), as both require precise control to maintain optimal deposition conditions [12]. In contrast, SbCl_5_, which exists in a liquid state with moderate volatility, exhibits a vapor pressure comparable to that of the EDOT monomer [12,40]. This similarity in vapor pressures between SbCl_5_ and monomer reactants allows for more precise control over process parameters, particularly in regulating reactant flow rates at different working pressures within the oCVD reactor [40].

### 4.6. Effect of Oxidant Saturation Ratio on Electrical Conductivity and Nanostructure in Conjugated Polymers

The oxidant saturation ratio (OSR) is a critical process parameter in the oCVD method, playing a key role in determining the doping level, lattice structure, and molecular organization of the deposited polymer thin films [12,39,40]. Specifically, OSR has a direct influence on the π-π stacking distance, which in turn affects the charge transfer integral and ultimately governs the electrical conductivity of the resulting conjugated conducting and semiconducting thin film [40]. OSR is mathematically defined as the ratio of partial pressure (P) to the saturation pressure ($P_{sat}$) of the oxidant at the designated deposition temperature [39,40]:

(10) $$OSR=\frac{P_{\left(oxidant\right)}}{P_{sat\;(oxidant)}}$$

The OSR determines the availability of oxidant species during polymerization, directly impacting polymer growth kinetics, oxidation state, and charge carrier density [40]. An optimal OSR ensures efficient doping, enhances thin film uniformity, and prevents unwanted side reactions, such as overoxidation or chlorination of conjugated backbone [40]. 

**Figure 8:**
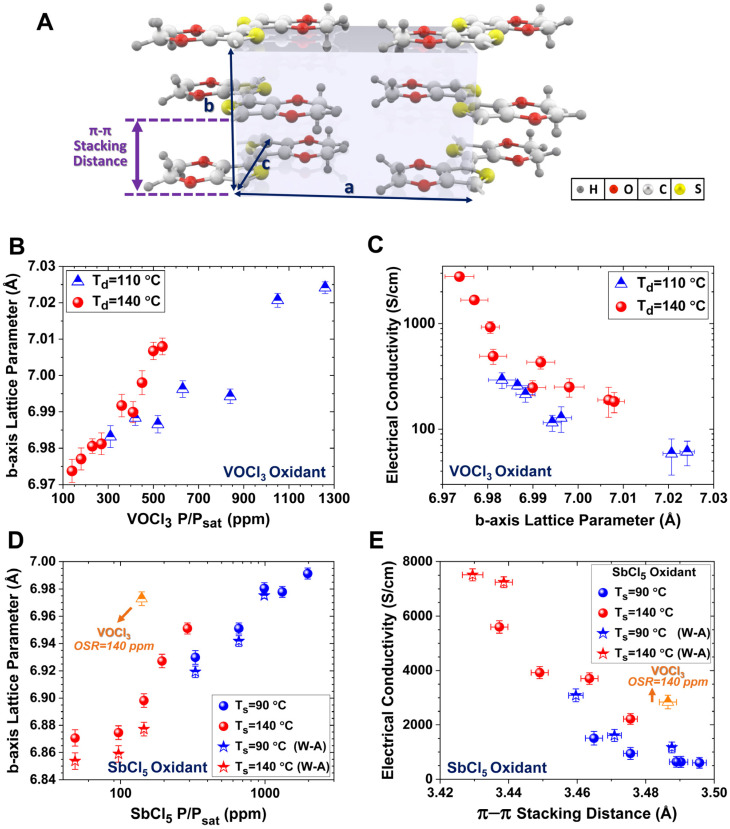
(**A**) Schematic of the orthorhombic unit cell of PEDOT, highlighting the b-axis lattice parameter and π-π stacking distance [40]. (**B**) Variation of the b-axis lattice parameter with VOCl_3_ saturation ratio [39]. (**C**) Relationship between electrical conductivity and b-axis lattice parameter in oCVD PEDOT thin films fabricated by the VOCl_3_ oxidant [39]. (**D**) Effect of oxidant saturation ratio on the b-axis lattice parameter in SbCl_5_-based oCVD PEDOT thin films [40]. (**E**) Electrical conductivity as a function of π-π stacking distance in SbCl_5_-based PEDOT films [40]. (**A**,**D**,**E**) Reproduced with permission [40]. Copyright 2021, Wiley. (**B**,**C**) Reproduced with permission [39]. Copyright 2019, Science.

In the orthorhombic structure of PEDOT, the π-π stacking distance corresponds to half of the b-axis lattice parameter, as illustrated in Figure 8A [40]. The OSR plays a crucial role in determining both the b-axis lattice parameter and π-π stacking distance of PEDOT polymer chains [5,39,40]. For oCVD PEDOT thin films fabricated using VOCl_3_ as the oxidant, an increase in OSR leads to an expansion of the b-axis lattice parameter, which is primarily due to higher dopant incorporation and chlorination of the conjugated backbone, which alters the molecular packing of the polymer chains (Figure 8B) [39]. The correlation between a reduced b-axis lattice parameter and enhanced electrical conductivity in VOCl_3_-based oCVD PEDOT thin films is presented in Figure 8C [39]. The polymer backbone of tie-chains aligns with the direction of high charge carrier mobility, facilitating efficient charge transport between crystallites. As the π-π stacking distance decreases, the charge transfer integral increases exponentially, leading to a significant boost in carrier mobility and electrical conductivity [5,30,39,40]. This highlights the critical role of molecular packing and dopant-induced structural modifications in optimizing the electrical performance of oCVD PEDOT thin films.

The incorporation of water-assisted (W-A) growth in the oCVD process leads to a smaller b-axis lattice parameter and consequently a reduced π-π stacking distance compared to films grown without W-A conditions, as shown in Figure 8D [40]. The reduction in π-π stacking distance while using W-A method in the oCVD process is attributed to improved PEDOT chain rearrangement, which results from more efficient oxidant decomposition, lower levels of non-ionized dopants, and the suppression of backbone chlorination [12,40]. The improved molecular packing in W-A oCVD PEDOT thin films enhances the interchain charge transfer integral, enabling faster charge transport, which in turn increases carrier mobility and electrical conductivity, as shown in Figure 8E [40].

The presence of oxidant by-products can severely disrupt the semi-crystalline structure of PEDOT thin films, leading to structural disorder and performance degradation [5,12,34,39]. The purity of oCVD-fabricated conductive polymer coatings is crucial, as oxidant by-products, contaminants, or unintended dopants can alter the nanostructure, affect electrical properties, and compromise the long-term stability of the films. To evaluate and mitigate these effects, X-ray photoelectron spectroscopy (XPS) is used to analyze the elemental composition and detect unwanted dopants [30,42]. Additionally, accelerated aging tests under varying environmental conditions (e.g., humidity, temperature, and UV exposure) are conducted to assess the long-term stability of the thin films.

When SbCl_5_ is used as an oxidant, its primary decomposition oxidant by-product is SbCl_3_, a volatile compound that off-gasses during deposition and desorbs from the substrate due to its relatively high vapor pressure (~1 Torr at 49 °C) [34,40]. To minimize the formation of oxidant by-products, such as SbCl_3_ and Sb^3+^ cations, in oCVD using liquid oxidants, it is essential to maintain high deposition temperatures and a low oxidant-to-monomer flow rate within the reactor [1,5,12,34,42]. Higher substrate temperatures and the implementation of water-assisted (W-A) oCVD have been shown to reduce chlorination in the conjugated backbone, limiting the presence of non-ionized chlorine species [30,40,42]. Simultaneously, ionic dopant incorporation in PEDOT thin films, particularly SbCl_6_^−^ and Cl^−^ species, increases with higher substrate temperatures and W-A processing, indicating improved ionized doping efficiency [30,40,42]. However, there exists an optimal level of ionized dopant incorporation, beyond which further doping does not improve electrical conductivity due to the scattering of charge carriers. Instead, excessive doping introduces ionized scattering centers, which negatively impact carrier mobility by increasing charge carrier scattering. This phenomenon is well-described by the Brooks–Herring–Dingle (BHD) model, which explains the trade-off between dopant incorporation and charge transport efficiency [5,12,25].

### 4.7. Effect of Deposition Temperature on Electrical Conductivity and Nanostructure in Conjugated Polymers

**Figure 9:**
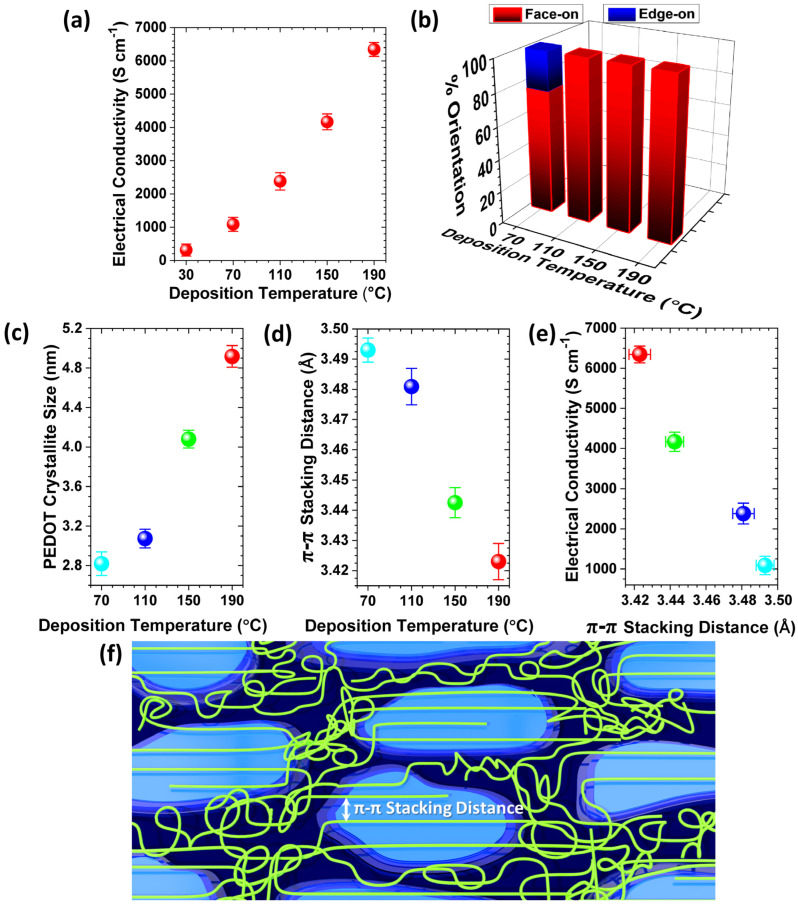
(**a**) Influence of deposition temperature on the electrical conductivity of oCVD PEDOT thin films [30]. (**b**) Variation in face-on and edge-on orientations with deposition temperature [30]. (**c**) Effect of deposition temperature on crystallite size [30]. (**d**) Correlation between the deposition temperature and π-π stacking distance [30]. (**e**) Relationship between the π-π stacking distance and electrical conductivity [30]. (**f**) Schematic of charge transport pathways, illustrating quasi-1D conductivity, where crystalline regions (light blue) exhibit higher conductivity, while amorphous regions (dark blue) are less conductive. The green lines represent the conjugated backbone chain, where the crystallite regions exhibit higher molecular ordering [30]. (**a**–**f**) Reproduced with permission [30]. Copyright 2024, Wiley.

Flexible substrates, such as polyethylene terephthalate (PET), polyethylene naphthalate (PEN), and their blends (PEN/PET), can tolerate temperatures up to approximately 150 °C [30,157]. Optimizing oCVD parameters, such as oxidant and monomer flow rates, allows the fabrication of PEDOT films using SbCl_5_ without the need for post-deposition acidic treatment, making it well-suited for flexible, temperature-sensitive, and solution-sensitive substrates [12]. Additionally, incorporating water vapor during deposition further enhances the performance of oCVD-deposited conducting polymers while maintaining a fully dry fabrication process [40]. The in-plane electrical conductivity of PEDOT-Cl thin films is strongly influenced by the deposition temperature, as illustrated in Figure 9a [30]. As the deposition temperature increases, electrical conductivity improves, reaching its peak value of 6345 ± 210 S/cm at 190 °C. This trend highlights the crucial role of temperature control in optimizing the electronic properties of oCVD PEDOT-Cl films [30].

The texture and nanostructure of conjugated conducting and semiconducting polymers play a crucial role in electrical conductivity, influencing charge carrier mobility and delocalization of charge carries [5,12,14,25,30,34]. In oCVD PEDOT thin films, semi-crystalline structures primarily develop when oxidant by-products are absent [12,30,39,40]. At low deposition temperatures (e.g., <70 °C), residual oxidant by-products lead to an amorphous morphology, whereas higher deposition temperatures facilitate by-product desorption, promoting semi-crystalline film formation [30]. Employing SbCl_5_ as a liquid oxidant at elevated deposition temperatures (e.g., >70 °C) is crucial for eliminating post-deposition acidic rinsing while promoting semi-crystalline film formation [30].

When the deposition temperature exceeds the glass transition temperature ($T_{g}$) of PEDOT (~100 °C) [5,39], crystallite reorientation occurs, minimizing interfacial energy and promoting a highly face-on orientation (Figure 9b) [30]. Higher deposition temperatures also lead to increased crystallite size (Figure 9c) by reducing deposition rates, enhancing precursor diffusion, and improving EDOT monomer accessibility to chloride dopants, facilitating the growth of larger crystallites [30].

The π-π stacking distance in PEDOT thin films decreases with rising deposition temperature (Figure 9d) [30]. This trend is attributed to dopant distribution, where low deposition temperatures promote C–Cl non-ionized dopant clustering and reduce ionized Cl^−^ incorporation, leading to undesirable backbone chlorination [30,34,42]. The combined effects of chlorination of conjugated backbone, dopant agglomeration, and presence of oxidant by-products at lower deposition temperatures result in an expanded π-π stacking distance within the orthorhombic crystallite structure of oCVD PEDOT thin films [30]. A reduced π-π stacking distance lowers the charge carrier hopping barrier, significantly enhancing electrical conductivity (Figure 9e) [30]. Since interchain charge transport is the rate-limiting factor, minimizing the π-π stacking distance improves the charge transfer integral, thereby increasing carrier mobility and electrical conductivity [5,6,30,39]. As the interchain charge transfer integral scales exponentially with decreasing π-π stacking distance, both carrier mobility and conductivity exhibit exponential enhancement [30,39,40].

The intermolecular interactions and polymer architecture play a critical role in determining crystallinity and amorphicity in conjugated polymers [5]. Crystalline regions emerge from strong p-orbital overlap, facilitating efficient charge delocalization, while amorphous regions result from weak interactions among disordered polymer chains [5,39]. High molecular weight polymers with densely packed, ordered structures and minimal sp^3^-carbon defects enable chains to bridge crystallites without significant conjugation loss [30,39,167]. A decreased π-π stacking distance imposes geometric constraints, increasing the probability of a single conjugated polymer chain bridging multiple crystallites, thereby promoting quasi-1D conductivity, as depicted in Figure 9f [30]. Charge transport occurs through two primary mechanisms: (i) intra-chain conduction across amorphous regions and (ii) interchain transport within crystalline domains [5,30]. Since interchain transport is inherently slower, in-plane conductivity is primarily governed by the charge transfer integral, which is highly sensitive to the π-π stacking distance and the b-axis lattice parameter [30,39,40].

### 4.8. Effect of Deposition Temperature on Carrier Mobility, Carrier Density, and Seebeck Coefficient 

**Figure 10:**
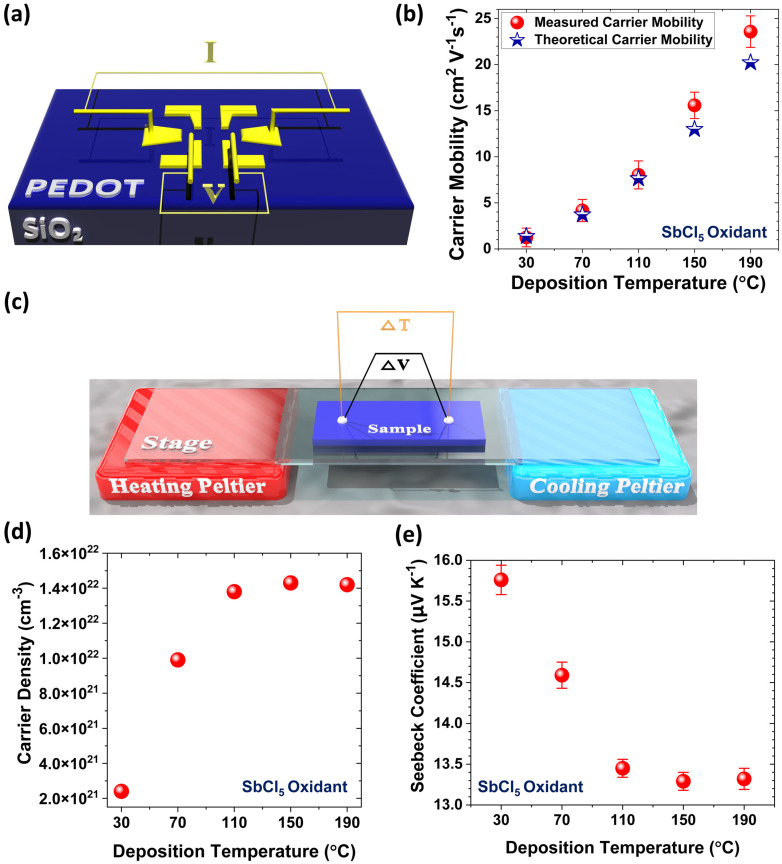
Comparison of theoretical and experimental carrier mobility in oCVD PEDOT thin films. (**a**) Schematic of the van der Pauw method in a cryogenic setup [30]. (**b**) Experimental and theoretical carrier mobility in PEDOT thin films as a function of the deposition temperature [30]. (**c**) Schematic of the Seebeck coefficient measurement setup, where Peltier plates create a temperature gradient, and temperature and voltage are measured simultaneously [30]. (**d**) Carrier density variation in PEDOT thin films with the deposition temperature [30]. (**e**) Seebeck coefficient of PEDOT thin films fabricated at different deposition temperatures [30]. (**a**–**e**) Reproduced with permission [30]. Copyright 2024, Wiley.

A comprehensive understanding of the electrical transport mechanism in conducting polymers requires measuring conductivity across a wide absolute temperature range, which can be achieved using the van der Pauw method in a cryogenic setup (Figure 10a) [30]. This method also determines carrier mobility and Hall effect parameters by analyzing resistance variations in thin films [30,168]. In conducting polymers, temperature-activated conductivity arises from disordered regions between crystallite domains, affecting charge transport behavior [5,30,41,169,170,171]. The increase in carrier mobility of conducting polymers with higher deposition temperatures (Figure 10b) [30], is linked to improved crystalline structure, including larger crystallite size, reduced π-π stacking distance, and a dominant face-on semi-crystalline orientation [5,12,30,40]. This interdependence of molecular packing, crystallinity, and electronic properties highlights the importance of process optimization in achieving high-performance conjugated polymers for electronic and energy applications [5,53].

The theoretical carrier mobility in conjugated polymers can be computed by incorporating the experimental Seebeck coefficient (measured using the setup in Figure 10c) [30] into the parabolic energy band diagram, where carrier mobility (*μ*) is determined using the following equation [30,172]:

(11) $$\mu =\sigma_{E0}\;\cdot \;{\left(\frac{m*}{m_{e}}\right)}^{-\frac{3}{2}}\;\cdot \;\frac{2\pi^{2}\hbar^{3}}{e\;(2m_{e}k_{B}T{)}^{\frac{3}{2}}}\;\cdot \;\frac{sF_{s-1}(\eta )}{F_{1/2}(\eta )}$$

where $\sigma_{E0}$ is the electrical transport coefficient, $m_{e}$ is the electron mass, m* is the effective charge carrier mass, ℏ is the reduced Planck constant, e is the elementary charge, $k_{B}$ is the Boltzmann constant, T is the absolute temperature, s is a model factor (typically s = 1 for PEDOT), $F_{i}(\eta )$ is the non-normalized Fermi-Dirac integral, and η is the reduced chemical potential.

The η value at ~300 K can be derived from the measured Seebeck coefficient (S) using the following equation [30,41,172]:

(12) $$S=\frac{k_{B}}{e}\;\cdot \;\left\{\frac{\left(s+1\right)\;F_{s}(\eta )}{s\;F_{s-1}(\eta )}-\eta \right\}$$

$F_{i}(\eta )$ can be computed using the following equation [30,173]:

(13) $$F_{i}\left(\eta \right)=\int_{0}^{\infty }\frac{\varepsilon^{i}}{1+e^{\varepsilon -\eta }}\;d\varepsilon$$

where e represents Euler’s number (the mathematical constant serving as the base of the natural logarithm), and ε serves as the variable of the integration.

The Equations (11)–(13) show that only charge carriers above the transport edge contribute to conductivity, while those below remain localized. Solving for the energy difference between the Fermi energy and mobility edge energy ($E_{f}-E_{t}$) allows modeling of η(T), capturing the temperature-dependent charge transport behavior more accurately in conducting and semiconducting polymers [30]. Both experimental and theoretical carrier mobility for PEDOT thin films increase with higher deposition temperatures (Figure 10a). The PEDOT thin film deposited at 190 °C with the use of SbCl_5_ oxidant shows the highest theoretical mobility of 20.22 cm^2^ V^−1^ s^−1^, closely matching the experimental Hall effect measurement of 23.58 ± 1.71 cm^2^ V^−1^ s^−1^ [30].

Doping plays a crucial role in enhancing conductivity in conjugated polymers. Oxidation (electron removal from HOMO) or reduction (electron addition to LUMO) significantly increases conductivity [6]. This process requires a strong oxidizing agent (p-type dopant) or a strong reducing agent (n-type dopant). Typically, 1/3 to 2/3 of monomer units are doped in conjugated polymers, corresponding to a doping concentration of 10^19^–10^22^ cm^−3^ [1,5,42]. The carrier density of oCVD PEDOT thin films strongly depends on the deposition temperature (Figure 10d) [30]. Studies show that the carrier density increases with the deposition temperature but stabilizes beyond a certain temperature (e.g., 110 °C). This rise is due to greater ionic counterion doping, which alters the structure and shape of the conjugated backbone [5,30,40]. Higher doping levels promote the benzoid-to-quinoid transition, extending conjugation lengths and improving charge delocalization [1,6,30,34,40]. 

Optimizing the thermoelectric power factor requires precise charge carrier density tuning due to its complex relationship with electrical conductivity and the Seebeck coefficient. The Seebeck coefficient (S) follows $S\sim \frac{m*}{n^{2/3}}$, where it depends on the carrier density (n) and effective mass (m*). In highly doped semiconductors, S is typically inversely proportional to n, as described by the single parabolic band (SPB) model [30,174,175,176]:

(14) $$S=\frac{8\pi^{2}k_{B}^{2}T}{3eh^{2}}\cdot m*\cdot {\left(\frac{\pi }{3n}\right)}^{\frac{2}{3}}$$

where $k_{B}$ is the Boltzmann constant, e denotes unit of charge, and h presents the Planck constant.

The consistent Seebeck coefficient in PEDOT thin films fabricated at a deposition temperature of 110 °C and above (Figure 10e) [30] indicates stable carrier density across these samples. Thus, the notable increase in carrier mobility is likely the key driver behind the enhanced electrical conductivity observed in PEDOT thin films deposited above 110 °C.

## 5. Electrical Conductivity of Conjugated Conducting Polymers

High electrical conductivity in conjugated polymers is generally achievable when quasi-1D conduction is present [5,6,30]. In conjugated conducting and semiconducting polymers, high electrical conductivity can be achieved under specific conditions: (i) large crystallite size, (ii) short π-π stacking distances to ensure high inter-chain charge transfer and prevent charge localization; (iii) a planar polymer backbone; (iv) a highly face-on or highly edge-on texture that offers improved percolation pathways and increased tie chains without orientation anisotropy, and (v) the formation of ultrathin films or fiber-like structures of the conjugated polymers [1,5,30,40].

**Figure 11:**
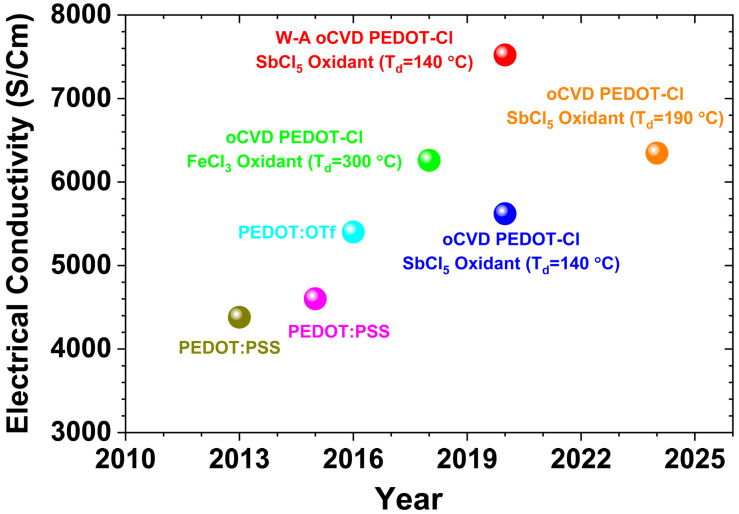
Timeline of electrical conductivity improvements in PEDOT thin films produced using different fabrication methods. Notably, the highest electrical conductivity, approximately 7520 S/cm, was achieved in PEDOT thin films fabricated via the water-assisted oCVD process using SbCl_5_ as a liquid oxidant at a deposition temperature (T_d_) of 140 °C.

The timeline of electrical conductivity improvements in various PEDOT thin films fabricated using different methods and counter-ion dopants, including PEDOT:PSS, PEDOT:OTf, and oCVD PEDOT-Cl, is exhibited in Figure 11. The highest reported conductivity of 7520 ± 240 S/cm was achieved using the water-assisted (W-A) oCVD method with SbCl_5_ as the oxidant, at a deposition temperature of 140 °C, by optimizing the π-π stacking distance and nanostructure of as-deposited PEDOT films without acidic rinsing [40]. Another reported high electrical conductivity in PEDOT thin films was about 6345 S/cm in as-deposited PEDOT-Cl fabricated by the oCVD method by the use of the SbCl_5_ oxidant and a deposition temperature of 190 °C [30]. PEDOT-Cl thin films fabricated at a deposition temperature of 300 °C with FeCl_3_ solid oxidant after HBr acidic rinsing demonstrated a conductivity of 6259 S/cm [41]. Additionally, as-deposited oCVD PEDOT-Cl thin films grown at a deposition temperature of 140 °C with the SbCl_5_ without water-assisted method yielded an electrical conductivity of 5602 S/cm [40]. Other notable electrical conductivity values include 5400 S/cm for PEDOT:OTf fabricated via in situ chemical polymerization (ICP) [177], 4380 S/cm for PEDOT:PSS nanofibrils with H_2_SO_4_ treatment [178], and 4600 S/cm for ultrathin PEDOT:PSS via solution-shearing [179].

## 6. Utilization of Conjugated Conducting Polymers on Flexible Optoelectronic Devices

**Figure 12:**
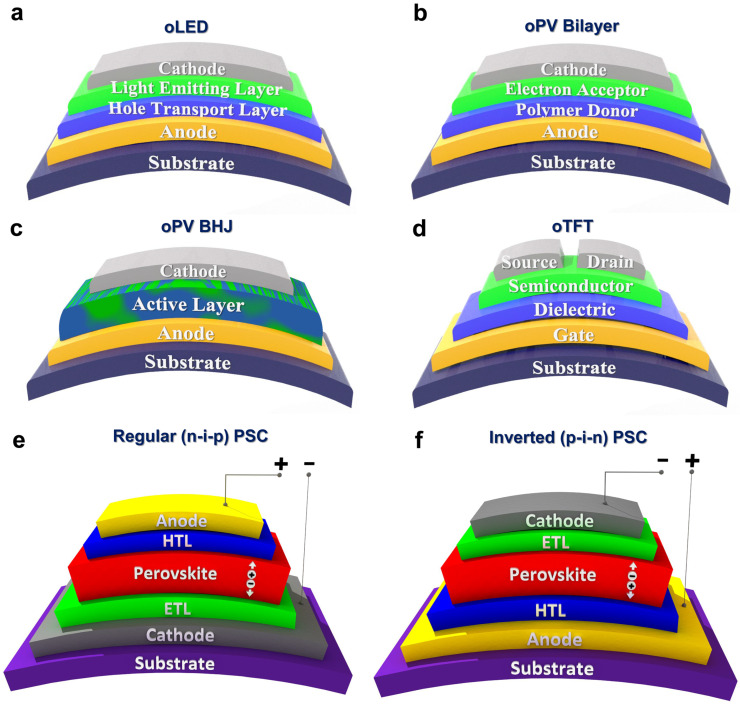
Schematics representations of flexible organic device structures based on conducting polymers. The schematic illustration featuring the integration of conducting and semiconducting conjugated polymers within one or more layers of the device stack in flexible optoelectronic devices. These include (**a**) organic light-emitting diodes (oLED), (**b**) organic photovoltaics (oPV) with a bilayer photoactive region, (**c**) oPV incorporating a bulk heterojunction (BHJ) photoactive layer, (**d**) organic thin-film transistors (oTFT) [1]. The flexible perovskite solar cells (PSC) with (**e**) regular structure (n-i-p) and (**f**) PSC with an inverted structure (p-i-n), where conducting polymer serves as the HTL. (**a**–**d**) Reproduced with permission [1]. Copyright 2021, Walter de Gruyter GmbH & Co KG.

Conjugated conducting and semiconducting polymers are attracting significant interest due to their low-cost fabrication, abundant raw materials, large-area scalability, mechanical flexibility, lightweight properties, and compatibility with flexible substrates [1,165]. Applications of conjugated conducting polymers include organic light-emitting diodes (OLEDs) for displays and lighting, organic photovoltaics (OPVs) based on both bilayer and bulk heterojunction configurations for solar energy conversion, and organic thin-film transistors (OTFTs) for flexible electronics and wearable sensors. Furthermore, these polymers are integral components in perovskite solar cells (PSCs), with both regular (n-i-p) and inverted (p-i-n) architectures, where they function as efficient hole transport layers (HTLs), enhancing charge extraction and device stability [34,39]. In inverted PSCs, the deposition order starts with the p-type layer, known as the HTL, being applied prior to the perovskite photoactive layer, followed by the deposition of the n-type layer, known as the electron transport layer (ETL) [34]. Interest in electronic and optoelectronic applications extends to organic solids composed of small, conjugated molecules due to their similar functionalities. The straightforward purification process of small molecules enables detailed exploration of fundamental exciton physics, which can often be translated to macromolecular solids, enhancing our understanding of both material classes. Their adaptability and excellent electrical properties enable the development of flexible and stretchable devices, paving the way for applications, such as foldable displays, electronic textiles, biomedical devices, and integrated energy storage systems. Figure 12 illustrates various flexible device configurations that utilize conducting and semiconducting polymers, underscoring their importance in advancing the field of flexible and wearable electronics.

In both small-molecule and polymeric organic optoelectronic materials, the arrangement of the π-π stacking distance plays a crucial role in determining charge carrier delocalization and electrical transport properties [12]. These stacked regions of conjugation, often referred to as chromophores, are key to the materials’ photophysical behavior [1,12]. In small, conjugated molecules, photophysics are influenced by their aggregate structures, a concept that has been extended to polymeric conducting and semiconducting polymers [1,12,180]. Precisely tuning the π-π stacking distance is essential for optimizing the performance of conjugated polymers. This involves careful control over fabrication parameters to strengthen inter-chain coupling by reducing the π-π stacking distance, thereby enhancing the overall optoelectronic performance of devices based on these materials [5,34].

The face-to-face stacking of conjugated regions, which promotes interchain coupling, is referred to as H-aggregates [1,12,181]. In contrast, the side-by-side arrangement of these regions is known as J-aggregates. Interestingly, single isolated polymer chains tend to display photoemission characteristics typical of J-aggregates [12]. In solid-state photocarrier generation, the balance between J-aggregates and H-aggregates depends on the specific polymer and the morphology induced by different fabrication processes. For example, P3HT thin films generally exhibit H-aggregate behavior, whereas P3HT whiskers display characteristics associated with J-aggregates [181].

Significant research efforts have focused on optimizing perovskite solar cells (PSCs) based on formamidinium (FA) lead triiodide (FAPbI_3_), including modifications to the active layer and electron/hole transport layers [34,39,182,183]. In optoelectronic devices, PEDOT thin films are commonly used as the hole transport layer (HTL), which requires not only high electrical conductivity and optical transparency but also energy levels alignment between HOMO level of PEDOT and the valence band of the photoactive layer to minimize carrier recombination for efficient operation [34]. Reducing the energy mismatch between the HOMO level of the HTL and the valence band of the photoactive layer facilitates efficient hole transfer, leading to improvements in open-circuit voltage and overall solar cell performance [34,184].

The work function of conjugated conducting polymers fabricated by the oCVD method can be tuned by adjusting the level of counterion dopants [3,34,185]. Typically, higher ionic chloride doping levels in oCVD PEDOT thin films result in an increased work function [30,34]. Furthermore, the high acidity of PEDOT:PSS, mainly due to the low pH (~1–2) of PSS, can lead to etching of the counter electrode and unfavorable interactions with the perovskite photoactive layer, resulting in increased leakage currents, trap-assisted charge recombination losses, and reduced long-term device stability [3,34,39,166]. In contrast, PEDOT thin films fabricated using the oCVD method demonstrate improved device stability compared to PEDOT:PSS-based devices [34,39].

## 7. Conclusions and Outlook

CVD methods have emerged as powerful manufacturing techniques for fabricating conjugated conducting and semiconducting polymer thin films. Unlike traditional solution-based processing, oCVD enables the deposition of high-purity, solvent-free, and conformal polymer layers, making it compatible with insoluble macromolecules, flexible substrates, and nanostructured surfaces. The absence of surface tension effects in oCVD prevents common issues, such as pinhole formation and film thickness variations, which are often encountered in liquid-based deposition methods. Furthermore, low processing temperatures (typically near room temperature) allow direct deposition on fragile, cost-effective substrates, such as paper, textiles, and plant leaves, without requiring complex transfer steps.

The ability to systematically tune the electrical, chemical, and mechanical properties of oCVD polymers has opened pathways for next-generation optoelectronic and energy devices. The combination of high electrical conductivity, ultra-thin film uniformity, and conformal deposition enables the fabrication of flexible, breathable electrodes for wearable sensors, thermoelectric, photovoltaics, supercapacitors, and batteries. Since the first demonstration of oCVD-based conducting polymer fabrication, significant progress has been made in expanding the portfolio of oCVD polymers. More than a dozen homopolymers have been reported, with copolymerization strategies now being developed to fine-tune film properties. Advances in oxidant selection, especially the use of volatile oxidants like SbCl_5_, have transformed oCVD into a true single-step, all-dry polymerization technique, eliminating the need for post-processing steps such as solvent rinsing. The formation of strong covalent bonds at the interface improves film adhesion, reducing the likelihood of delamination-induced device failure. This interfacial control has also enabled the fabrication of subtractive lithographic patterns with nanoscale resolution (<100 nm), crucial for microelectronic applications.

Advancing texture and nanostructure engineering in conjugated conducting and semiconducting polymers presents a promising strategy for tuning carrier mobility, electrical conductivity, ionic conductivity, and optoelectronic properties. Key parameters, such as doping level, π-π stacking distance, intra- and inter-chain interactions, crystallite connectivity, grain boundaries, and crystallite size, must be precisely controlled to optimize metal–insulator transition behavior. Achieving high carrier mobility while maintaining high carrier density remains a significant challenge, as improper nanostructure control can lead to charge localization and hinder transport efficiency. The charge transfer integral, which dictates electronic coupling strength, plays a crucial role in enhancing electrical conductivity at the microscopic scale. Future research should focus on fine-tuning these structural parameters through advanced fabrication techniques, ensuring controlled molecular ordering to further improve the performance of conducting polymers in electronic and energy applications.

Despite its success at the laboratory scale, transitioning oCVD to industrial production remains an important challenge. However, recent reports have demonstrated the feasibility of large-scale roll-to-roll oCVD processing, paving the way for commercial applications. Unlike high-temperature vacuum-based techniques that require film transfer from a sacrificial substrate, oCVD’s low-temperature processing enables direct deposition onto flexible substrates, eliminating costly and complex transfer steps. The scalability, precision, and material versatility of oCVD position it as a promising next-generation manufacturing platform for flexible electronics, energy storage, and wearable devices. Future research efforts will likely focus on further expanding the application of oCVD polymers, optimizing deposition parameters, and integrating oCVD films into commercial device architectures.

The oCVD method continues to push the boundaries of conducting polymer fabrication, offering a solvent-free, highly scalable, and conformal deposition technique suitable for electronic and energy applications. Recent advancements, particularly in liquid oxidants, control of oxidant saturation ratio, and nanostructure engineering, have demonstrated capability of oCVD method to produce ultrathin, defect-free coatings with tunable electrical and morphological properties. Fine-tuning doping levels and optimizing π-π stacking distances have further improved charge transport, paving the way for higher conductivity and enhanced device performance.

One exciting avenue for future research is the exploration of novel liquid oxidants beyond those recently developed, which could further enhance metallic conductivity at lower deposition temperatures. While metallic-like behavior has been observed in oCVD-fabricated conductive polymers at higher deposition temperatures, achieving this at reduced deposition temperatures would improve compatibility with flexible substrates, significantly expanding material versatility and industrial applicability. Furthermore, integrating real-time in situ nanostructure characterization techniques, such as in situ X-ray diffraction, will be essential for monitoring and controlling polymer crystallinity and molecular ordering during deposition, ensuring optimized charge transport properties and enhanced film performance.

The roll-to-roll (R2R) oCVD process marks a transformative step toward high-throughput, large-area manufacturing of conducting and semiconducting polymers. As an environmentally friendly and cost-effective alternative to conventional deposition methods, R2R oCVD has the potential to revolutionize flexible electronics, wearable sensors, energy storage devices, and optoelectronic applications. However, further research is needed to precisely control polymerization kinetics, optimize oxidant delivery, and develop real-time process monitoring to ensure uniform film properties on a large scale. By addressing these challenges, oCVD can solidify its role as a next-generation, sustainable manufacturing approach, bridging the gap between laboratory-scale research and industrial-scale production. Its seamless integration with emerging technologies, such as printed electronics and hybrid nanomanufacturing, could establish oCVD as a foundational method for the next era of functional thin film materials.

## Figures and Tables

**Table 1:** Common precursors utilized and materials fabricated in various CVD methods. This table summarizes the typical precursors used and common functional materials deposited via different CVD techniques, including 2D materials, conducting polymers, semiconductors, dielectrics, superconductors, and protective coatings.

CVD Method	Typical Reactants	Common Materials Fabricated
**CVD for 2D Materials**	Hydrocarbon Gases (Methane, Acetylene, Ethylene, Propane) + Hydrogen	Graphene Films (Monolayer and Multilayer) [4,47,68]
**Transition Metal Dichalcogenides (TMDs) Growth via CVD**	Metal Precursors (e.g., MoO_3_, WO_3_, Nb_2_O_5_, MoCl_5_, WCl_6_) + Chalcogen Precursors (S, Se, H_2_S, H_2_Se)	TMDs [4,48,49,50,51,52]
**Oxidative CVD (oCVD)**	Oxidant (e.g., FeCl_3_, VOCl_3_, SbCl_5_) + Monomer(s) (e.g., EDOT, pyrrole, aniline)	Conducting Polymers [3,12,30,40,53,69], Semiconducting Polymers [3,7,53]
**Initiated CVD (iCVD)**	Radical Initiator (e.g., TBPO) + Monomer(s) (e.g., acrylate, methacrylate)	Functional Polymers [9,10,64,70,71]
**Metal-Organic CVD (MOCVD)**	Metal-Organic Precursors (e.g., TMGa, TEGa, TMA, TMIn, Y(thd)_3_) + Reactant Gases (O_2_, NH_3_, NH_3_, PH_3_, AsH_3_)	Metal oxides [4,72,73], III-V Compound Semiconductors [4,58,74,75,76], High Temperature Superconductors [59,62,63,77,78]
**Plasma-Enhanced CVD (PECVD)**	Volatile precursor (s) (e.g., SiH_4_, CH_4_, CF_4_, TEOS) + Plasma Activation	Dielectric Thin Films [79,80]
**Low-Pressure CVD (LPCVD)**	Gaseous precursors (e.g., SiH_4_, NH_3_, SiCl_2_H_2_, B_2_H_6_, PH_3_) + Reactant Gases (O_2_, N_2_O)	Semiconductors [4,81,82], Dielectric [4,83], Protective Coatings [4,84]

Abbreviation used in the table: molybdenum trioxide (MoO_3_), tungsten trioxide (WO_3_), niobium pentoxide (Nb_2_O_5_), molybdenum pentachloride (MoCl_5_), tungsten hexachloride (WCl_6_), sulfur (S), selenium (Se), hydrogen sulfide (H_2_S), hydrogen selenide (H_2_Se), 3,4-ethylenedioxythiophene (EDOT), iron chloride (FeCl_3_), vanadium oxytrichloride (VOCl_3_), antimony pentachloride (SbCl_5_), tert-butyl peroxide oxide (TBPO), trimethylgallium (TMGa), triethylgallium (TEGa), trimethylaluminum (TMA), trimethylindium (TMIn), and yttrium(III) tetramethylheptanedionate (Y(thd)_3_), ammonia (NH_3_), phosphine (PH_3_), arsine (AsH_3_), silane (SiH_4_), methane (CH_4_), tetrafluoromethane (CF_4_), tetraethyl orthosilicate (TEOS), dichlorosilane (SiCl_2_H_2_), diborane (B_2_H_6_), nitrous oxide (N_2_O).

**Table 2:** Summary of key advantages and disadvantages of various fabrication methods for conjugated conducting and semiconducting polymers. Various factors, including thin film quality, conformality, substrate compatibility, and process scalability, are summarized for different fabrication methods.

Method	Deposition Type	Conformality	Substrate-Independent	Key Advantages	Key Disadvantages
**Spin-coating**	Solution-Based	Poor	Highly Dependent	Simple, Cost-Effective,	Limited to Soluble Polymers, Lacks Conformal Coating, Incompatible with Solution-Sensitive Substrates, Substrate-Dependence; Restricted to Planar Substrates; Ineffective for Complex Surfaces, Low Material Utilization (Only 2–5% of Polymer Solution), Low-Quality Thin Films with Contaminants
**In Situ Chemical Polymerization (ICP)**	Solution-Based	Low	Highly Dependent	Simple, Cost-Effective, Well-Dispersed Nanoparticles; Ideal for Composites	Lacks Conformal Coating, Unsuitability for Solvent-Sensitive Substrates, Potential for Flocculant Formation
**Electrochemical Polymerization**	Solution-Based	Moderate	Highly Dependent	Simple, Cost-Effective, Excellent Substrate Adhesion	Substrate-Dependence; Requires Conductive Substrates, Incompatible with Solution-Sensitive Substrates,
**Vapor Phase Polymerization (VPP)**	Mixed of Solution- and Vapor-Based	Moderate	Moderate Dependent	Operation in a Moderate Vacuum Environment, Fabrication of Smooth Thin Films, Good Quality of Thin Films, Excellent Substrate Adhesion	Process Complexity, Incompatible with Solution-Sensitive Substrates; Requires Oxidant Pre-Coating, Limited Scalability, High Cost for Batch Reactor
**Oxidative Chemical Vapor Deposition (oCVD)**	Vapor-Based	High	Largely Independent	Highly Conformal Coating, Substrate Independent, High Quality Thin Films, High Control Over Thin Film Properties, Ability to Create Ultrathin Films (<10 nm), Low to Moderate Deposition Temperature, Excellent Substrate Adhesion, Low-Cost in R2R Process, and Compatibility with Large-Scale Production, Industrial Method	Process Complexity, High Cost for Batch Reactor

**Table 3:** Overview of common oxidants in conjugated conducting polymer fabrication. This table highlights widely used oxidants in the synthesis of conjugated conducting polymers, covering both solution-based and vacuum-based fabrication techniques [2]. Reproduced with permission [2]. Copyright 2024, Royal Society of Chemistry.

Fabrication Method	Explored Oxidants
Spin-coating	Polymer is typically dissolved in a solution, with FeCl_3_ occasionally added as oxidant
Electropolymerization	Oxidation of a monomer is induced by voltage or current
In Situ Chemical Polymerization (ICP)	FeCl_3_, CuCl_2_, Cu(ClO_4_)_2_, Fe(pTS), Fe(Tos)_3_, Ammonium peroxydisulfate (APS)
Vapor Phase Polymerization (VPP)	FeCl_3_, CuCl_2_, Fe(OTs)_3_, Fe(OTf)_3_
Oxidative Chemical Vapor Deposition (oCVD)	FeCl_3_, CuCl_2_, Br, VOCl_3_, SbCl_5_
